# Association between Subcortical Morphology and Cerebral White Matter Energy Metabolism in Neonates with Congenital Heart Disease

**DOI:** 10.1038/s41598-018-32288-3

**Published:** 2018-09-19

**Authors:** Nina Gertsvolf, Jodie K. Votava-Smith, Rafael Ceschin, Sylvia del Castillo, Vince Lee, Hollie A. Lai, Stefan Bluml, Lisa Paquette, Ashok Panigrahy

**Affiliations:** 1 Keck School of Medicine, University of Southern California, Los Angeles, CA USA; 2 Department of Pediatrics, Division of Cardiology, Children’s Hospital of Los Angeles, Los Angeles, CA USA; 3 Department of Pediatric Radiology, Children’s Hospital of Pittsburgh of UPMC and University of Pittsburgh School of Medicine, Pittsburgh, USA; 4 Department of Biomedical Informatics, University of Pittsburgh School of Medicine, Pittsburgh, USA; 5 Department of Anesthesiology, Critical Care Medicine Children’s Hospital of Los Angeles, Los Angeles, CA USA; 6 Department of Radiology, Children’s Hospital of Los Angeles, Los Angeles, CA USA; 7 Department of Pediatrics, Division of Neonatology, Children’s Hospital of Los Angeles, Los Angeles, CA USA

## Abstract

Complex congenital heart disease (CHD) is associated with neurodevelopmental impairment, the mechanism of which is unknown. Cerebral cortical dysmaturation in CHD is linked to white matter abnormalities, including developmental vulnerability of the subplate, in relation to oxygen delivery and metabolism deficits. In this study, we report associations between subcortical morphology and white matter metabolism in neonates with CHD using quantitative magnetic resonance imaging (MRI) and spectroscopy (MRS). Multi-modal brain imaging was performed in three groups of neonates close to term-equivalent age: (1) term CHD (n = 56); (2) preterm CHD (n = 37) and (3) preterm control group (n = 22). Thalamic volume and cerebellar transverse diameter were obtained in relation to cerebral metrics and white matter metabolism. Short echo single-voxel MRS of parietal and frontal white matter was used to quantitate metabolites related to brain maturation (n-acetyl aspartate [NAA], choline, myo-inositol), neurotransmitter (glutamate), and energy metabolism (glutamine, citrate, creatine and lactate). Multi-variate regression was performed to delineate associations between subcortical morphological measurements and white matter metabolism controlling for age and white matter injury. Reduced thalamic volume, most pronounced in the preterm control group, was associated with increased citrate levels in all three group in the parietal white matter. In contrast, reduced cerebellar volume, most pronounced in the preterm CHD group, was associated with reduced glutamine in parietal grey matter in both CHD groups. Single ventricle anatomy, aortic arch obstruction, and cyanotic lesion were predictive of the relationship between reduced subcortical morphometry and reduced GLX (particularly glutamine) in both CHD cohorts (frontal white matter and parietal grey matter). Subcortical morphological associations with brain metabolism were also distinct within each of the three groups, suggesting these relationships in the CHD groups were not directly related to prematurity or white matter injury alone. Taken together, these findings suggest that subplate vulnerability in CHD is likely relevant to understanding the mechanism of both cortical and subcortical dysmaturation in CHD infants. Future work is needed to link this potential pattern of encephalopathy of CHD (including the constellation of grey matter, white matter and brain metabolism deficits) to not only abnormal fetal substrate delivery and oxygen conformance, but also regional deficits in cerebral energy metabolism.

## Introduction

Neonates with congenital heart disease (CHD) are known to be at risk for impaired neurodevelopmental outcomes, which is likely related to an interplay of cerebral dysmaturation and acquired brain injury, including white matter injury^1^. Recent animal model and correlative neuropathological studies suggest that cortical dysmaturation is linked to white matter abnormalities, including developmental vulnerability of the subplate in CHD^2,3^. Recent neuroimaging studies have documented subcortical morphological abnormalities in CHD patients across the lifespan^4–8^, but the relationship between subcortical morphological abnormalities and subplate/white matter vulnerability in CHD is unknown, despite this link being well-established in preterm infants^9–12^.

Here, we used quantitative multi-modal magnetic resonance imaging (MRI) and magnetic resonance spectroscopy (MRS) in infants with CHD to test the hypothesis that subcortical morphological measurements are associated with selective metabolic cerebral white matter alterations. We focused on thalamic and cerebellar morphological measurements as these subcortical structures are potential mediators of poor neurodevelopmental outcomes via important etiological factors including poor cerebral substrate delivery and genetic alterations^4–7,13–15^. We used quantitative short echo MRS, which allows for measurement of parietal and frontal white matter metabolites related to brain maturation (n-acetyl aspartate [NAA], choline, and myo-inositol), neurotransmitters (glutamate) and energy metabolism (glutamine, citrate, creatine, lactate)^12,16–21^. Given the known vulnerability of the subplate in preterm infants, we delineated the relationship between subcortical morphology and white matter metabolism in term CHD infants relative to two preterm cohorts (with and without CHD), controlling for white matter injury. We hypothesized that: (1) reduced subcortical morphology would be associated with global alterations in cerebral white matter maturation related metabolites (reduced NAA, elevated myo-inositol and choline) representing global metabolic brain dysmaturation similar to premature infants; (2) reduced subcortical morphology would be associated with selected alterations in cerebral white matter energy metabolites (creatine, lactate, citrate, glutamine) in the presence of punctate white matter lesions.

## Methods

### Patient Recruitment and Clinical Data Collection

Neonates with CHD (term and preterm) undergoing clinically indicated MRI scans at Children’s Hospital Los Angeles were recruited between 2002 and 2010. Inclusion criteria included severe forms of CHD expected to require corrective or palliative cardiac surgery during infancy. Patients were scanned at approximately term-equivalent gestational age either in the pre-operative or post-operative period. We also included a comparison group of preterm infants without CHD who were recruited from a high-risk NICU at the same institution as previously published^12,17,18,20^. Subjects were enrolled after the clinically indicated MRI was acquired. The clinical indications for the brain MRI for the CHD groups included: (1) focal neurological abnormality (including seizures) (25%);(2) concern for hemorrhage or brain injury (50%); (3) concern for brain dysgenesis (25%).

With regards to timing of the clinical MRI relative to surgery: (1) approximately 1/3 of the scans were pre-operative and 2/3 post-operative for both the preterm CHD and term CHD cohorts; (2) there was greater variation in the timing of the pre-operative MRI scan between the preterm CHD and term CHD cohorts. For example, the pre-operative MRI scan for the preterm group was performed between 1 week to 4 months of time of surgery (depending on stability of the preterm CHD neonates for surgery and the severity of the heart lesion) compared to most of the pre-operative term CHD cases in which the pre-operative MRI was performed in the first week of life before surgery. The post-operative scan for both of the preterm CHD and term CHD groups in our study were all performed at least within 52 weeks corrected postconceptional age.

Exclusion criteria included major congenital brain malformations. All MRIs were completed under direction of the primary clinical care team. Clinical characteristics were obtained via chart review. Risk Adjusted Congenital Heart Surgery Score (RACHS-1) and Aristotle score of complexity were calculated^22,23^.

This study was approved by the institutional review board (IRB) of Children’s Hospital of Los Angles and written informed consent was obtained from each subject’s parent or legal guardian except for a few patients in which MRI was obtained clinically, and retrospective use of the data was approved by the IRB protocol. All experiments were performed in accordance with the institutional guidelines and regulations.

### Neonatal Brain MRI and MRS protocol

MRI studies were acquired under clinical indications on a GE 1.5T (Signa LX, GE Healthcare, Milwaukee, WI) MR system using a custom-built neonatal transmit-receive head coil. Conventional imaging studies were acquired with the MRS studies and included a 3D coronal SPGR sequence (TE = 6 ms; TR = 25 ms, FOV = 18 cm; matrix = 256 × 160; slice thickness 1.5 mm, spacing 0 mm) or axial and sagittal T1-weighted FLAIR sequences (TE = 7.4, TR = 2100; TI = 750; FOV = 20 cm; Matrix = 256 × 160), axial T2-weighted FSE sequence (TE = 85 ms, TR = 5000 ms, FOV = 20 cm, matrix = 320 × 160 or 256 × 128) and a diffusion-weighted sequence (TE = 80; TR = 10000; FOV = 22 cm; Matrix = 128 × 128; slice thickness = 4.5 mm, spacing 0 mm). The 3D T1-weighted, T2-weighted, and diffusion-weighted images were reviewed by two pediatric neuroradiologists (HAL and AP) for evidence of punctate white matter lesion, hypoxic-ischemic injury, acute focal infarction, and hemorrhage as previously described^24^.

**Figure 1:**
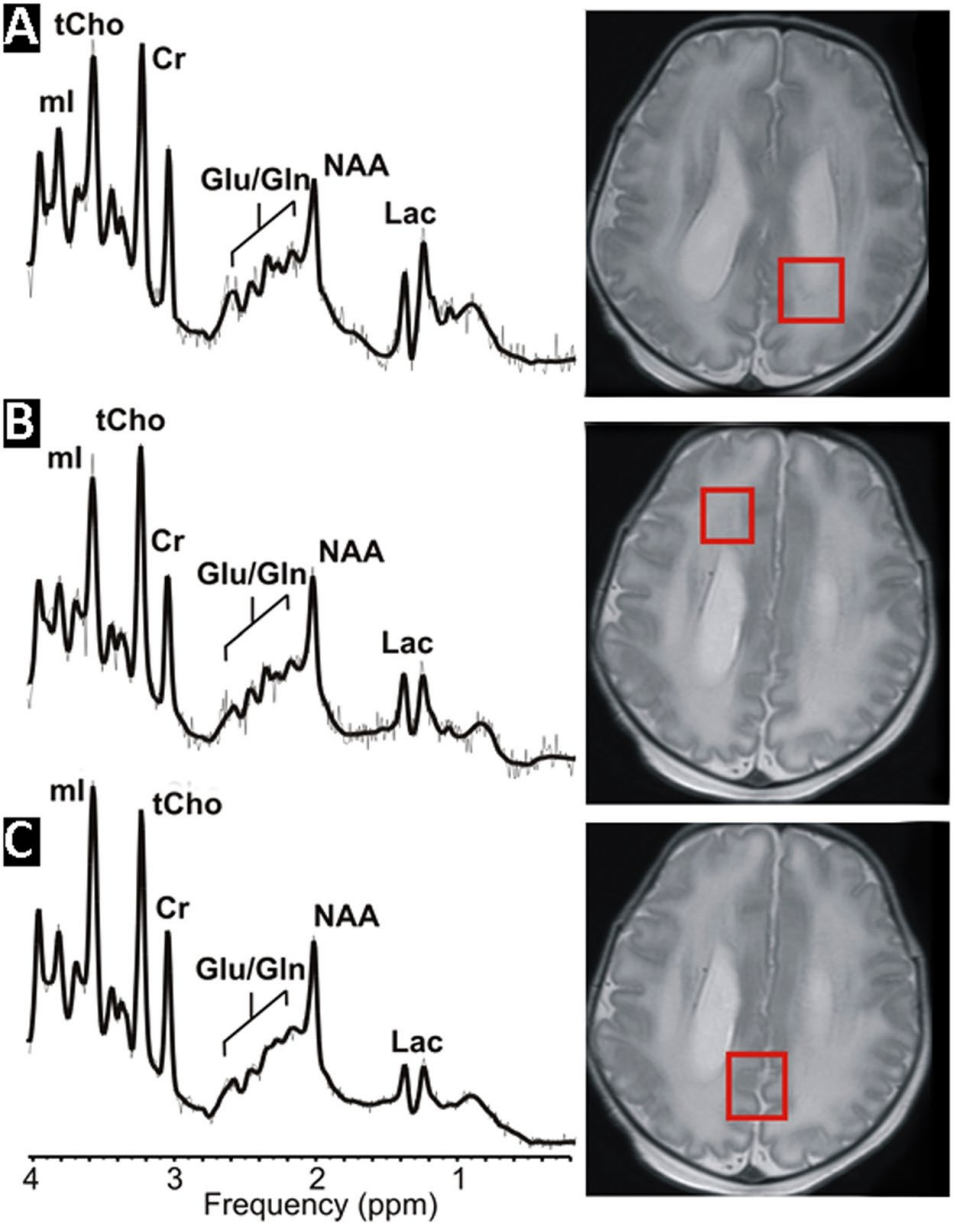
Quantitative MR Spectroscopy using short echo technique in a term neonate with congenital heart disease (CHD) (HLHS). Short echo technique allows quantitation of additional metabolites (myo-inositol [mI], glutamate [Glu], glutamine [Gln], above and beyond long echo technique, which can measures N-acetyl asparatate [NAA], Choline [Cho], and Creatine [Cr]). Three voxel location were obtained in the order of frequency: (top row) parietal white matter, (middle row) frontal white matter, and (bottom row) parietal-occipital grey matter.

With regards to MR spectroscopy acquisition, 1H spectra were acquired from a single voxel (approximately 3 cm^3^) placed in the developing parietal white matter dorsolateral to the trigone of the lateral ventricle in the left hemisphere using a point resolved spectroscopy (PRESS) sequence with a short echo time (TE) of 35 milliseconds (ms), a repetition time (TR) of 1.5 seconds, 128 signal averages, and a total acquisition time for each spectrum of approximately five minutes, including scanner adjustments. The parietal and frontal white matter locations were selected because (1) the parietal and frontal white matter is known to be a region of vulnerability in preterm infants; (2) numerous developing thalamocortical and corticocortical association pathways traverse that region^10,25^. (3) these white matter locations are known to demonstrate vulnerability in recent animal models of congenital heart disease (Fig. 1)^2,3^. For comparative purpose, a grey matter voxel was placed in the parietal –occipital midline region as previously described^12,16–18,20,21^.

### Thalamic and Cerebellar Morphological Assessment

**Figure 2:**
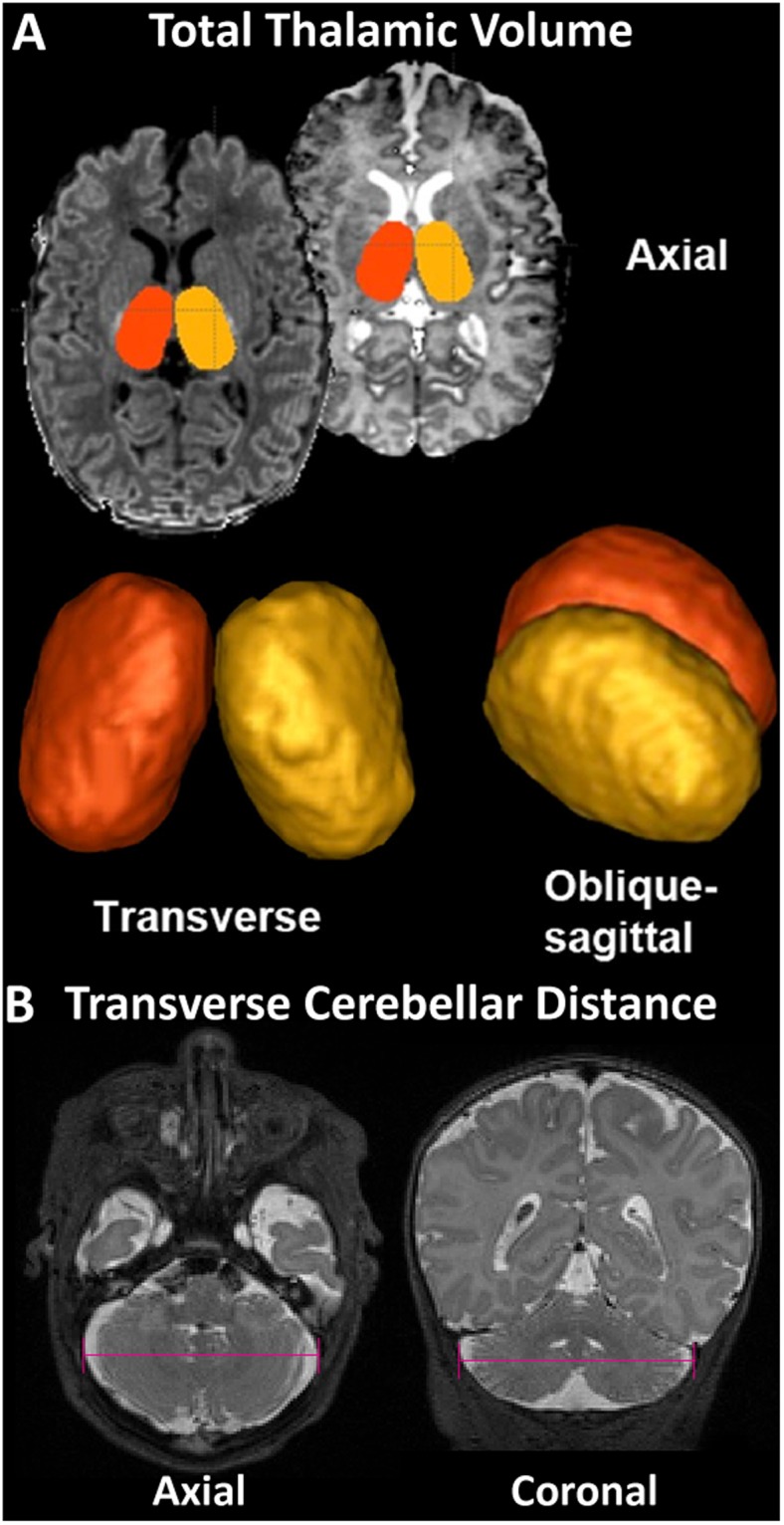
Subcortical morphology measurement methodology: (**A**) Quantitation of Thalamic Volume and (**B**) Cerebellar Transverse Diameter metric.

Bilateral regions of the thalamus were manually traced on the 3D coronal SPGR images by one person (RC) under the supervision of a senior pediatric neuroradiologist (AP) using ITK-SNAP as previously described (Fig. 2)^12,26^. The margins of the neonatal thalamus were determined with reference to a standard neuroanatomical atlas, the Stereotactic Atlas of the Human Thalamus and Basal Ganglia by Anne Morel. Axial T2 and coronal 3D SPGR images were co-registered when possible to aid in placing the contours. Inter (second rater: AP) and intra-rater reliabilities were assessed in a subset of cases (n = 5) and determined to be approximately 0.85 and 0.93^12,26^. To assess for cerebellar morphological abnormalities, we used the maximum transverse cerebellar hemisphere distance (diameter) as has been previously described and validated in infants with both prematurity and CHD (Fig. 2)^8,27–32^.

### Brain Metric Measurements

To further quantify the relationship of thalamic volume and transverse cerebellar volume to metrics of global brain volume, we obtained quantitative brain metric measurements of cerebral brain parenchyma and CSF (extra-axial and intra-axial/intraventricular), which was previously described for similar preterm and CHD infants and validated with outcome studies^8,27–32^. Specific cerebral brain paraenchymal measures included: (1) bifrontal lobe diameter; (2) left and right frontal lobe height; (3) brain and bone biparietal diameter, and (4) frontal-occipital lobe diameter. The extra-axial CSF measures of the pericerebral space included: (1) the interhemispheric distance; and (2) craniocaudal left and right interopercular distances. The intra-axial/intraventricular CSF measures included: (1) diameters of the left and right lateral ventricles and (2) the third ventricle. The MR images were displayed via a DICOM browser (DicomWorks, http://dicom.online.fr/) and these brain metrics were manually measured on four selected sections by one person (RC)^8,27–32^. Interobserver correlation from repeating measurements was calculated on 7 different scans by 3 observers. Intraobserver correlation was calculated and then rechecked to assess for variability^8,27–32^.

### MRS Data Processing

Metabolite concentrations were quantitated with fully automated LCModel software (Stephen Provencher, Inc., Ontario, Canada, version V6.13-1 C). This software automatically applies zero-order and first-order corrections for phase, estimates a baseline, and corrects for ppm shift and eddy currents as needed. Data fitting was determined from the linear combination of model spectra of known concentration, which included metabolites indicative of: (1) brain maturation (NAA, total choline, and myo-inositol); NAA is stored within neurons and axons and thought to be a measure of neuronal-axonal integrity; myo-inositol is thought to be a marker of immature myelination and an indicator of glial cells as part of signaling pathways as precursor for the phosphoinositide second messenger system; (2) neurotransmitters (glutamate); and (3) energy metabolism (glutamine, citrate, total creatine, lactate). For quantitation, the unsuppressed water signal was used as a concentration reference, with tissue water content estimated at a standardized value (86%) based on published reference data as previously described by our group^33,34^. Spectral quality was assessed by reviewing the signal-to-noise (SNR) the full width at half maximum (FWHM), which are reported by the processing software. Only spectra with SNR > 5 and FWHM <0.08ppm (5.1 Hz) were included in the analysis. Examples of 1H-MRS spectra voxel locations are provided in Fig. 2.

### Statistical Analyses

Our primary analysis was to examine the relationship between subcortical morphology and white matter metabolism. We defined our “exposure” or independent variable as thalamic volume/cerebellar transverse diameter and the “metabolic outcome measure” or dependent variable as the MRS metabolites measured with the parietal and frontal white matter voxels. This analysis was performed using multi-variate regression and three major co-variates were included in the model: postconceptional age (gestational age plus postnatal age), at time of MRI scan timing of scan relative to cardiac surgery (pre vs post-operative), presence of punctate white matter injury (PWMI). Multi-variate regression with false discovery rate (FDR) correction was used, to correct for multiple comparisons. The FDR is one way of conceptualizing the rate of type 1 errors in null hypothesis testing when conducting multiple comparisons. The grey matter metabolite data was also analyzed for relative regional comparison to the two white matter voxels. We also did an ad hoc analysis to delineate the influence of heart lesion subtypes on our primary outcomes measures: (1) MRS outcomes variables, (2) the relationship between subcortical morphology and white matter metabolism using the multi-variate analysis. Two major co-variates were included in the model: postconceptional age (gestational age plus postnatal age) and heart lesion subtype. We used three heart lesion subtype classification; (1) single vs double ventricle anatomy; (2) cyanotic vs acyanotic; (3) aortic arch obstruction vs no obstruction.

Our secondary analysis examined the relationship between subcortical morphology and more global cerebral structural measures of brain tissue parenchyma and CSF using the same modeling. We defined our “exposure” or independent variable as thalamic volume/cerebellar transverse diameter and the “cerebral structural outcome measure” or dependent variable as brain metrics grouped into six categories: [(1) total frontal: frontal diameter plus bilateral frontal height; (2) bi-parietal brain tissues diameter; (3) fronto-occipital diameter; (4) total extra-axial CSF (see above); (5) total intraventricular CSF (see above); (6) total CSF (extra-axial plus intraventricular)]. Our tertiary analysis was used to compare differences in clinical variable and subcortical measurements between the three clinical cohorts: (1) term CHD; (2) preterm CHD and (3) preterm without CHD. Clinical variables were compared using a three-way ANCOVA (analysis of covariance). We then compared each of the groups to each other using pairwise T-tests.

## Results

### Clinical Characterization of CHD cohorts

There were 93 CHD subjects that met the inclusion criteria for this study: 56 full term neonates with CHD and 37 preterm neonates with CHD (born 25–36 weeks), as well as 22 preterm control patients (born 24–36 weeks). Table 1 includes subject demographic information. Brain MRIs were obtained at similar post-menstrual ages for both preterm and term CHD groups (p = 0.4), and the infants in both CHD groups were predominantly male **(**Table 1**)** and had a similar spectrum of complex CHD (Table 2). There was no statistically significant difference in the rate of brain injury between pre-and post-operative imaging time points. Approximately 1/3 of the MRI scans were pre-operative and 2/3 of the MRI scan were post-operative in both the term and preterm CHD groups. The incidence of brain injury in the term CHD cohort was: hemorrhage 8%; infarct 8%; hypoxic-ischemic brain injury 5%; and punctate white matter injury 28%. The incidence of brain injury in the preterm CHD cohort was hemorrhage 8%; infarct 11%; hypoxic-ischemic brain injury 3%; and punctate white matter injury 19%. There was no statistical difference between the incidence of brain injury between the term CHD and preterm CHD groups relative to the pre-operative and post-operative scans (p = 0.087). There was also no difference in gender between the three cohorts (p = 0.297).

**Table 1:** Demographic Information of the CHD (Congenital Heart Disease) Cohort.

	Preterm CHD	Term CHD	Preterm control	p-value
	mean ± SD or N(%)	mean ± SD or N(%)	mean ± SD or N(%)	
Male	70.3%	62.5%	52.4%	0.44
Gestational Age (weeks)	32.4 ± 3.3	38.9 ± 1.2	29.3 ± 5.0	<0.001
Post-menstrual age at MRS scan (weeks)	43 ± 8	43 ± 4	44.8 ± 3.7	0.4

^*^CHD – Congenital Heart Disease.

**Table 2:** Cardiovascular Lesions Subtypes and Severity in CHD cohorts.

	Preterm CHD*	Term CHD
	mean ± SD or N(%)	mean ± SD or N(%)
RACHS-1 score^†^	3.5 ± 1.7	3.5 ± 1.3
Arisotole score	8.6 ± 4.0	8.6 ± 3.2
Single ventricle CHD	10 (27%)	12 (21%)
Cyanotic CHD	20 (54%)	29 (51%)
d-Transposition of Great Arteries	5 (13.5%)	5 (8.8%)
Isolated VSD^‡^	8 (22%)	8 (14%)
Non-Critical Aortic stenosis, VSD, Coarctation	2 (5.4%)	0
Isolated Coarctation	1 (2.7%)	5 (8.8%)
Aortic Arch Anomaly with VSD	5 (13.5%)	10 (17.5%)
Ebstein’s anomaly	2 (5.4%)	0
HLHS^§^	8 (21.6%)	8 (14%)
Other Single Ventricle	2 (5.4%)	7 (12.3%)
Tetralogy of Fallot	2 (5.4%)	5 (8.8%)
Truncus Arteriosus	1 (2.7%)	1 (1.8%)
Abnormal Pulmonary Artery Origins	1 (2.7%)	0
Critical Aortic Stenosis	0	3 (5%)
AV^||^ septal defect	0	1 (1.8%)
Partial Anomalous Pulmonary Venous Return	0	2 (3.5%)
Aortopulmonary Window	0	1 (1.8%)

*CHD – congenital heart disease.

^†^RACHS-1 Score – risk adjustment for congenital heart surgery.

^‡^VSD – ventricular septal defect.

^§^HLHS – hypoplastic left heart syndrome.

^||^AV–atrioventricular.

### Subcortical Morphological Measurements

**Figure 3:**
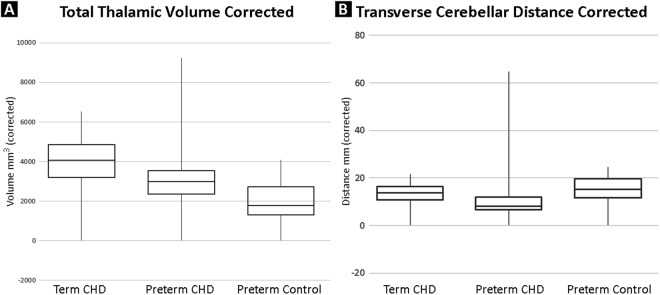
Morphological comparison between three groups: Compared to term CHD cohort, thalamic volume was reduced in both preterm CHD and preterm controls cohorts, with the preterm control cohort demonstrating the smallest thalamic volume (p-value < 0.0005) (**A**). In contrast, transverse cerebellar diameter was reduced in preterm CHD cohort compared to the term CHD and preterm control cohort (p value < 0.0001) (**B**).

Compared to term CHD cohort, thalamic volume was reduced in both preterm CHD and preterm controls cohorts, with the preterm control cohort demonstrating the smallest thalamic volume (p-value < 0.0005) (Fig. 3A). In contrast, transverse cerebellar diameter was reduced in preterm CHD cohort compared to the term CHD and preterm control cohort (p value < 0.0001) (Fig. 3B).

### Thalamic Morphology and Cerebral Parietal White Matter Metabolism Associations

Reduced thalamic volume was associated with increased citrate levels (energy metabolism) in the parietal white matter of the preterm CHD group (p < 0.0187, FDR-corrected) and preterm control group (p < 0.0105, FDR-corrected) (Table 3). We noted a marginally significant association between reduced thalamic volume and increased citrate levels in the parietal white matter of the term CHD group (p < 0.072, FDR-corrected) (Table 3). We did note associations between reduced thalamic volume and increased creatine (p < 0.0235, FDR-corrected, preterm CHD), and increased citrate (p < 0.0079, FDR-corrected, term CHD) that related to the presence of punctate white matter injury.

**Table 3:** Thalamic Volume and Parietal White Matter Metabolite Levels (Punctate White Matter Injury- PWMI covariate).

Metabolite	Term CHD*^†^					Preterm CHD^‡^					Preterm Control^§^			
	p-value	Direction	R^2^	Coeff Var	PWMI	p-value	Direction	R^2^	Coeff Var	PWMI	p-value	Direction	R^2^	Coeff Var
NAA	0.0524	+	0.456749	30.7418	0.7508	0.5178	−	0.877788	19.85294	0.3182	0.9513	−	0.492655	14.74062
Ins	0.2646	+	0.114891	18.89605	0.1372	0.9456	−	0.109775	23.45854	0.3688	0.9874	−	0.288581	12.98116
Cho	0.3967	−	0.110571	15.95958	0.8347	0.4602	−	0.138749	15.47945	0.1519	0.2556	−	0.078114	12.69787
Cr	0.105	+	0.227444	18.37348	0.3711	0.3859	−	0.734812	12.28451	**0**.**0235**	0.6885	+	0.414986	9.083397
Cit	0.072	−	0.360594	34.38952	**0**.**0079**	**0**.**035**	−	0.562449	34.41699	0.683	**0**.**0105**	−	0.56885	46.29472
Glx	0.6945	+	0.070643	36.25	0.205	0.3744	−	0.314752	24.62558	0.2405	0.5068	−	0.23159	21.85133
Gln	0.7948	−	0.090605	51.97592	0.066	0.1871	−	0.118248	35.06018	0.4546	0.6395	−	0.103319	27.48782
Glu	0.2812	+	0.178473	42.94807	0.8138	0.921	+	0.551101	31.20413	0.3043	0.5064	−	0.55102	23.70414
Lac	0.8579	−	0.108788	96.97419	0.3997	0.3846	+	0.140385	94.40527	0.4758	0.4317	−	0.050059	84.56339

*CHD – congenital heart disease.

^†^Term CHD N = 48.

^‡^Preterm CHD N = 33.

^§^Preterm Control N = 21.

Significant p-values bolded.

### Thalamic Morphology and Cerebral Frontal White Matter Metabolism Associations

We noted a marginally significant association between reduced thalamic volume and increased lactate (p < 0.0709, FDR-corrected) in the frontal white matter of the preterm CHD group (Table 4). We did note associations between reduced thalamic volume and reduced NAA (p < 0.0334, FDR-corrected); choline (p < 0.0205, FDR-corrected), and creatine (p < 0.039, FDR-corrected) in the term CHD group that were related to the presence of white matter injury.

**Table 4:** Thalamic Volume and Frontal White Matter Metabolite Levels (Punctate White Matter Injury- PWMI covariate).

Metabolite	Term CHD*^†^					Preterm CHD^‡^					Preterm Control^§^			
	p-value	Direction	R^2^	Coeff Var	PWMI	p-value	Direction	R^2^	Coeff Var	PWMI	p-value	Direction	R^2^	Coeff Var
NAA	0.9224	+	0.774584	18.24458	**0**.**0334**	0.7303	−	0.768441	23.95784	0.5873	0.717	+	0.760213	11.43802
Ins	0.5185	−	0.07478	22.94549	0.3266	0.8703	+	0.207865	18.06865	0.7881	0.4285	−	0.363603	14.70338
Cho	0.1554	−	0.424488	18.55258	**0**.**0205**	0.3431	−	0.064063	19.58286	0.8447	0.4316	−	0.193516	15.95949
Cr	0.435	−	0.569085	18.15663	**0**.**039**	0.4645	−	0.606128	16.01159	0.8237	0.5449	−	0.377854	8.559466
Cit	0.3077	+	0.250202	48.92149	0.9982	0.6837	−	0.269977	50.8386	0.5444	0.5512	+	0.311292	100.1061
Glx	0.6174	−	0.25446	30.33791	0.3343	0.9715	+	0.235047	20.45129	0.5849	0.1391	−	0.513777	11.67991
Gln	0.5506	−	0.079573	39.31749	0.2562	0.4774	+	0.041081	39.61548	0.8802	0.7464	−	0.0524	21.92894
Glu	0.835	−	0.459397	33.98833	0.6959	0.4651	−	0.392674	35.05339	0.4267	0.3723	−	0.277317	30.38067
Lac	0.9712	−	0.246979	78.11362	0.7549	0.0709	+	0.322697	81.33639	0.7574	0.336	−	0.371781	106.5916

*CHD – congenital heart disease.

^†^Term CHD N = 48.

^‡^Preterm CHD N = 33.

^§^Preterm Control N = 21.

Significant p-values bolded.

### Thalamic Morphology and Cerebral Parietal-Occipital Grey Matter Metabolism Associations

For regional comparison, we also examined the relationship between thalamic volume and parietal/occipital grey matter metabolism (Table 5). We did note associations between reduced thalamic volume and reduced choline (p < 0.025, FDR-corrected) in the term CHD group that was related to the presence of punctate white matter injury.

**Table 5:** Thalamic Volume vs Grey Matter Metabolite Levels (Punctate White Matter Injury-PWMI covariate).

Metabolite	Term CHD*^†^					Preterm CHD^‡^					Preterm Control^§^			
	p-value	Direction	R^2^	Coeff Var	PWMI	p-value	Direction	R^2^	Coeff Var	PWMI	p-value	Direction	R^2^	Coeff Var
NAA	0.2682	+	0.513689	25.07823	0.151	0.5117	−	0.777316	24.38283	0.2692	0.4995	+	0.551257	17.02956
Ins	0.1797	−	0.241566	20.46368	0.1058	0.5925	−	0.51165	17.75506	0.6181	0.7183	+	0.518071	12.33746
Cho	0.1436	−	0.19551	21.86055	**0**.**025**	0.9104	+	0.055695	21.45106	0.3206	0.6731	−	0.018364	19.32098
Cr	0.9538	−	0.215358	12.45477	0.2278	0.7755	−	0.33546	18.65613	0.1125	0.9231	−	0.230641	13.06458
Cit	0.443	+	0.130406	46.01967	0.512	0.9106	−	0.419624	51.3946	0.3046	0.2138	−	0.431148	40.96013
Glx	0.9371	−	0.144025	55.65984	0.8597	0.67	+	0.179859	41.27373	0.2849	0.6453	+	0.144443	38.19435
Gln	0.4491	−	0.078472	80.24896	0.4192	0.3066	+	0.073915	54.12166	0.2827	0.4391	+	0.041052	43.88989
Glu	0.2723	+	0.300859	44.44031	0.2966	0.7783	−	0.378798	41.15354	0.4148	0.893	+	0.297378	42.3951
Lac	0.1623	−	0.08102	95.24125	0.2487	0.4531	+	0.028973	128.6598	0.5878	0.4085	−	0.287103	134.2488

*CHD – congenital heart disease.

^†^Term CHD N = 48.

^‡^Preterm CHD N = 33.

^§^Preterm Control N = 21.

Significant p-values bolded.

### Cerebellum Morphology and Cerebral Parietal White Matter Metabolism Associations

Reduced transverse cerebellar diameter was associated with increased parietal white matter choline (p < 0.0367, FDR-corrected) in the term CHD group and increased parietal white matter myo-inositol (p < 0.0124, FDR-corrected) in the preterm controls (Table 6), independent of white matter injury. Reduced transverse cerebellar diameter was associated with reduced parietal white matter NAA levels (p < 0.0001, FDR-corrected) in the preterm CHD group (Table 6). We did note associations between reduced transverse cerebellar diameter and increased citrate (p < 0.0029, FDR-corrected, term CHD), and reduced creatine (p < 0.00124, FDR-corrected, preterm CHD) related to the presence of punctate white matter injury.

**Table 6:** Cerebellar Distance vs Parietal White Matter Metabolite Levels (Punctate White Matter Injury- PWMI covariate).

Metabolite	Term CHD*^†^					Preterm CHD^‡^					Preterm Control^§^			
	p-value	Direction	R^2^	Coeff Var	PWMI	p-value	Direction	R^2^	Coeff Var	PWMI	p-value	Direction	R^2^	Coeff Var
NAA	0.6407	−	0.416136	31.87021	0.4958	**0**.**0017**	+	0.90989	17.04723	0.2813	0.7744	+	0.494923	14.70764
Ins	0.9175	+	0.070756	19.36143	0.2293	0.2269	−	0.163331	22.74196	0.256	0.0124	−	0.502288	10.85774
Cho	0.0367	−	0.175722	15.36394	0.6634	0.6687	+	0.12396	15.61178	0.0949	0.1989	−	0.096767	12.56875
Cr	0.5419	−	0.173221	19.00733	0.6085	0.2362	+	0.737064	12.23223	**0**.**0124**	0.6072	−	0.418437	9.056564
Cit	0.3631	−	0.354099	34.56374	**0**.**0029**	0.1028	−	0.542143	35.20652	0.2004	0.1907	−	0.386541	42.53579
Glx	0.4651	−	0.072573	36.21234	0.2488	0.5259	+	0.301153	24.86874	0.1541	0.4536	+	0.236877	21.77602
Gln	0.4511	−	0.100698	51.68668	0.0625	0.7741	−	0.068676	36.03224	0.228	0.176	+	0.18215	26.25175
Glu	0.7932	−	0.158784	43.45966	0.6456	0.1652	+	0.57581	30.33319	0.3546	0.9285	+	0.539767	23.99934
Lac	0.228	−	0.112919	96.74916	0.4447	0.1479	−	0.170891	92.71498	0.3785	0.5266	−	0.038192	85.08993

*CHD – congenital heart disease.

^†^Term CHD N = 48.

^‡^Preterm CHD N = 33.

^§^Preterm Control N = 21.

Significant p-values bolded.

### Cerebellum Morphology and Cerebral Frontal White Matter Metabolism Associations

Reduced transverse cerebellar diameter was associated with increased levels of frontal white matter myo-inositol (p < 0.0237, FDR-corrected) in the term CHD group (Table 7). Reduced transverse cerebellar diameter was associated with reduced frontal white matter NAA (p < 0.0003, FDR-corrected) and increased lactate (p < 0.002, FDR-corrected) in the preterm CHD cohort. Reduced transverse cerebellar diameter was associated with reduced frontal white matter choline (p < 0.0282, FDR-corrected), reduced NAA (p < 0.0325, FDR-corrected), and reduced creatine (p < 0.0398, FDR-corrected) in the term CHD group, related to the presence of white matter injury.

**Table 7:** Cerebellar Distance vs Frontal White Matter Metabolite Levels (Punctate White Matter Injury- PWMI covariate).

Metabolite	Term CHD*†					Preterm CHD‡					Preterm Control^§^			
	p-value	Direction	R^2^	Coeff Var	PWMI	p-value	Direction	R^2^	Coeff Var	PWMI	p-value	Direction	R^2^	Coeff Var
NAA	0.4918	−	0.778018	18.1051	**0**.**0325**	**0**.**0168**	+	0.821545	21.03203	0.8254	0.9758	−	0.751202	11.65094
Ins	0.0237	−	0.264797	20.45401	0.3097	0.5805	+	0.215972	17.97595	0.93	0.4399	−	0.358151	14.76623
Cho	0.0523	−	0.45451	18.0622	**0**.**0282**	0.3362	+	0.068871	19.53249	0.7358	0.169	−	0.43594	13.347
Cr	0.0847	−	0.610024	17.27263	**0**.**0398**	0.1811	+	0.629437	15.53059	0.827	0.6172	−	0.356999	8.701743
Cit	0.9997	+	0.218816	49.93494	0.9015	0.1407	−	0.326118	48.84468	0.8823	0.2423	−	0.481916	86.82464
Glx	0.8105	−	0.249692	30.43476	0.3746	0.4648	+	0.261354	20.09656	0.4911	0.7386	−	0.128662	15.63562
Gln	0.6313	−	0.076826	39.37612	0.2932	0.6335	−	0.026901	39.90732	0.9863	0.1529	−	0.450369	16.70093
Glu	0.8915	+	0.457514	34.04747	0.7243	0.1715	+	0.430649	33.93981	0.4308	0.7829	+	0.114513	33.62905
Lac	0.949	−	0.247291	78.09743	0.7531	**0**.**0118**	−	0.421547	75.16704	0.6151	0.6226	+	0.238319	117.3693

*CHD – congenital heart disease.

^†^Term CHD N = 48.

^‡^Preterm CHD N = 33.

^§^Preterm Control N = 21.

Significant p-values bolded.

### Cerebellum Morphology and Cerebral Parietal-Occipital Grey Matter Metabolism Associations

For comparison, we also examined the relationship between cerebellar volume and parietal/occipital grey matter metabolism (Table 8). We did note an association between reduced transverse cerebellar diameter and reduced glutamine (p < 0.0413, FDR-corrected) in the term CHD group and reduced transverse cerebellar diameter and reduced glutamine, (p < 0.0062, FDR-corrected) and glutamine/glutamate (p < 0.0107, FDR-corrected) in the preterm CHD.

**Table 8:** Cerebellar Distance vs Gray Matter Metabolite Levels (Punctate White Matter Injury- PWMI covariate).

Metabolite	Term CHD*^†^					Preterm CHD^‡^					Preterm Control^§^			
	p-value	Direction	R^2^	Coeff Var	PWMI	p-value	Direction	R^2^	Coeff Var	PWMI	p-value	Direction	R^2^	Coeff Var
NAA	0.1659	+	0.514248	25.06381	0.0799	0.3659	+	0.780517	24.20692	0.2617	0.069	+	0.626574	15.53484
Ins	0.5734	−	0.189181	21.15861	0.1763	0.3077	−	0.530256	17.41354	0.4283	0.6433	−	0.520681	12.30401
Cho	0.617	−	0.134178	22.67855	0.0512	0.0883	+	0.129681	20.59359	0.4715	0.536	−	0.031256	19.19368
Cr	0.2136	+	0.24051	12.25353	0.1667	0.3814	+	0.34497	18.52215	0.1321	0.8679	+	0.231551	13.05685
Cit	0.7947	−	0.122417	46.23058	0.4939	0.8926	+	0.418349	51.45103	0.3207	0.5406	−	0.386541	42.53579
Glx	0.1532	−	0.229157	52.81952	0.9246	**0**.**0107**	+	0.320524	37.56786	0.5325	0.6599	+	0.143438	38.21678
Gln	0.0413	−	0.193067	75.09379	0.6527	**0**.**0062**	+	0.229741	49.35872	0.6735	0.6514	+	0.01635	44.45159
Glu	0.9565	+	0.282702	45.01368	0.3234	0.086	+	0.431421	39.37189	0.5183	0.7345	+	0.301754	42.26287
Lac	0.3122	−	0.079001	95.34581	0.1554	0.3209	−	0.040823	127.8723	0.6272	0.927	−	0.255409	137.2006

*CHD – congenital heart disease.

^†^Term CHD N = 48.

^‡^Preterm CHD N = 33.

^§^Preterm Control N = 21.

Significant p-values bolded.

### Influence of Cardiac Lesion Subtypes on the Relationship Between Subcortical Morphology and Metabolism in CHD

First, we noted that single ventricle, aortic arch obstruction and cyanotic heart lesion diagnosis were predictive of reduced GLX (particularly glutamine metabolism) in frontal white matter and parietal grey matter in both the term CHD and preterm CHD group (Supplemental Tables S1–S9). Next, our analysis demonstrated a consistent finding that single ventricle, aortic arch obstruction and cyanotic lesion predicted the relationship between reduced subcortical morphology metrics (thalamus/cerebellum) and reduced GLX (mostly glutamine metabolism) in both CHD cohorts in frontal white matter and parietal grey matter (Supplemental Tables S12–S27).

### Subcortical and Cerebral Morphological Associations in CHD groups

We correlated the subcortical measurements with cerebral measurements of brain parenchyma and CSF volume (Tables 9 and 10). In regards to our secondary analysis, reduced thalamic volume and reduced transverse cerebellar diameter were correlated with reduced global cerebral brain parenchymal metrics (frontal total distance, biparietal diameter and fronto-occipital distance) in the term CHD cohort (p < 0.0001, FDR-corrected) (Tables 9 and 10). A similar relationship was identified in the preterm control group (Tables 9 and 10). Only reduced transverse cerebellar volume (p < 0.0001, FDR-corrected), but not thalamic volume, was correlated with brain parenchymal measurements in the preterm CHD group. No relationship was seen between thalamic volume and CSF volume in the term CHD and preterm control group (Tables 9 and 10). In contrast, the preterm CHD group demonstrated increased transverse cerebellar diameter with increased volume of CSF. Of note, there were no significant relationships between subcortical measurement and cerebral brain metrics that were related to the presence of punctate white matter injury.

**Table 9:** Relationship Between Thalamic and Cerebral Morphologic Metrics in CHD and Preterm Control.

	Term CHD*			Preterm CHD			Preterm Controls	
	N used	Thalamic Volume	PWMI^†^	N used	Thalamic Volume	PWMI	N used	Thalamic Volume
		p-value	p-value		p-value	p-value		p-value
Frontal volume	49	0.0093	0.9114	34	0.0807	0.2765	22	0.022
Biparietal diameter	49	<0.0001	0.5719	35	0.058	0.2251	22	<0.0001
Fronto-occipital diameter	49	<0.0001	0.2455	35	0.9658	0.9176	22	0.0148

*CHD – congenital heart disease.

^†^PWMI- punctate white matter injury (presence/absence) was included as a co-variate (except in the preterm cohort which did not have WMI).

**Table 10:** Relationship Between Cerebellar and Cerebral Morphologic Metrics in CHD and Preterm Controls.

	Term CHD*			Preterm CHD			Preterm Controls	
	N used	Cerebellar Distance	PWMI^†^	N used	Cerebellar Distance	PWMI	N used	Cerebellar Distance
		p-value	p-value		p-value	p-value		p-value
Frontal volume	49	0.0049	0.9109	34	0.0002	0.9696	22	0.8151
Biparietal diameter	49	<0.0001	0.3145	35	0.0003	0.7783	22	0.001
Fronto-occipital diameter	49	<0.0001	0.4071	35	0.1785	0.8629	22	0.0008

*CHD – congenital heart disease.

^†^PWMI- punctate white matter injury (presence/absence) was included as a co-variate (except in the preterm cohort which did not have WMI).

## Discussion

Recent animal models of CHD suggest that abnormal cerebral cortical development is secondary to cerebral white matter abnormalities, likely reflecting developmental vulnerability of the developing subplate^2,3^. In parallel, recent CHD neuroimaging studies have delineated important subcortical morphological reductions that are not only present in the fetal and preoperative neonatal period, but are also persistent across the lifespan and likely mediate poor neurocognitive outcomes^4–8^. These subcortical abnormalities are also likely related to cerebral white matter abnormalities and subplate vulnerability, and these relationships have been well established in preterm infants^9–12^. However, the relationship between subcortical morphology and white matter/subplate vulnerability has not been studied in detail in infants with CHD. Hypoxia associated with congenital heart disease reduces proliferation and neurogenesis in the subplate structures and developing white matter, thus reducing long-term growth of both cortical and likely also subcortical structures^2,3^.

Here, for the first time, we delineate *in vivo* associations between subcortical (thalamic and cerebellum) morphological reductions and altered cerebral white matter metabolism in both preterm and term CHD infants. We had anticipated that these subcortical morphological reductions, which were also associated with other global measures of cerebral structures (as measured with brain metrics), would be associated with global alterations in brain maturation metabolites (NAA, choline and myo-inositol) which normally change dramatically with age during the time period of our study. Reduced thalamic volume, most pronounced in the preterm control group, was associated with increased citrate levels in all three group in the parietal white matter. In contrast, reduced cerebellar volume, most pronounced in the preterm CHD group, was associated with reduced glutamine in parietal grey matter in both CHD groups. Single ventricle, obstructed aortic arch, and cyanotic types of CHD lesions were predictive of the relationship between reduced subcortical morphometry and reduced GLX (mostly glutamine) in both CHD cohorts (frontal white matter/parietal grey matter). Subcortical morphological associations with brain metabolism were distinct within each of the three groups, suggesting these relationships in the CHD groups were not directly related to prematurity or white matter injury alone.

Short echo quantitative MRS can measure multiple brain metabolites that are biomarkers for not only brain maturation, but also various energy processes (lactate, creatine, citrate, and glutamine). Most studies in CHD patients up to this point have used long-echo techniques, which is more limited in the spectrum of energy metabolites that can be measured (citrate and glutamine). Citrate is an intermediate in the tricarboxylic acid (TCA) cycle and accumulates in tissue where the glycolytic rate exceeds the TCA cycle activity^35^. Citrate can be used by cells to transport mitochondrial acetyl-CoA carbons to the cytoplasm for the biosynthesis of fatty acids ultimately needed for the *de novo* synthesis of cell membranes to support cell division important for neurogenesis^36^. Glutamine serves as a major precursor to glutamate in synapses^37,38^ More recently, however, glutamine has been implicated in a neuroprotective role and also serves as an energy metabolite^39^. Interestingly, we had also anticipated that the relationship between subcortical morphology and energy metabolites would be dependent on the presence of white matter injury, which was the case for some of the associations. However, in our study we found presence of white matter injury contributed little variance to the associations between reduced subcortical morphology and altered energy metabolites, including that of citrate and glutamine.

Term CHD infants are known to have delayed brain maturation similar to non-CHD preterm infants as documented by morphological studies relative to healthy controls. We had anticipated that reduced thalamic and cerebellar volume would be associated with globally altered brain maturation metabolites based on previously published normative references (namely reduced NAA, increased choline, and increased my inositol) in term CHD infants. While reduced subcortical volume was associated with reduced overall cerebral growth as reflected in our brain metric analysis, we did not see a similar global alteration in relation to brain maturation metabolites. The only relationship between reduced subcortical volume and metabolic brain dsymaturation was between reduced cerebellum volume and increased myo-inostiol in the frontal white matter in term CHD. Myo-inositol was also inversely related to thalamic volume in the preterm control group. Myo-insoitol is a precursor to phosphatidylinositol, a membrane phospholipid involved in signal transduction. It is present in the white matter prior to active myelination and acts as a buffer in regulating extracellular osmolarity. Given that myo-inositol is dramatically decreasing with age during the last half of gestation and around birth^20^, coinciding with the emergence of increased volume in the cerebellum and thalamus, these results like reflect a component of brain dysmaturation which is likely related to immature white matter development and delayed myelination in the CHD groups.

We also found that NAA in the cerebellum was positively correlated with thalamic volume in the preterm CHD group. NAA is produced in the mitochondria of neurons, then diffuses along the axoplasma and is degraded in oligodendrocytes^34^. Because NAA is stored in mature neurons and axons, its concentration is a good measure of the number and density of fully developed neurons in the brain. An NAA decrease is associated with neuronal injury and loss^34^. It is possible that some of these relationships that included altered NAA, may also potentially indicate a deficit in mitochondrial function, instead of just neuronal/axonal loss, which would be concordant with our finding related to citrate, creatine, and glutamine as described above. To this end, we have recently described novel findings regarding alterations in phosphocreatine— an important energy metabolite not previously described in CHD infants in one of our more recent studies performed at 3T which allows measurements of phosphocreatine^19^. In normal brain metabolism, phosophocreatine stores energy and maintains ATP levels and, thus is an important indicator of energy reserves as well as a potential marker of mitochondrial dysfunction^40^. As such, there may be a fundamental dysregulation of energy metabolism related to genetic and subtle hypoxic-ischemic risk factors in CHD. In addition, the connection between risk factors and both NAA and/or phosphocreatine raises the novel possibility of energy metabolism of mitochondrial brain dysfunction in CHD patients, which would need future studies to elucidate. These findings are also concordant with the proposed oxygen conformance changes that have been hypothesized to occur in the fetal brain of CHD subjects secondary to chronic or prolonged reduced cerebral substrate or oxygenation deficiency^41^.

Of note. thalamic volumes were lowest in the preterm controls and cerebellar diameter reduced in preterm CHD, suggesting a differential regulation of subcortical development, We hypothesize that the thalamic volume was the lowest in the preterm control group because of a relatively higher vulnerability for acquired diffuse periventricular white matter injury and associated thalamic dysmaturation relative to the CHD groups. The preterm control group has been used in other preterm related analyses that we have previously published^18^, and even though there was no evidence of focal punctate white matter lesion or moderate/severe DEHSI, this preterm control group was recruited from a high risk NICU. As such this preterm cohort has a high incidence of important clinical risk factors for diffuse white matter injury including sepsis (50%), 33% chronic lung disease (33%) and necrotizing enterocolitis (25%). Interestingly, the preterm control group did demonstrate: (1) a relationship between reduced thalamic volume and elevated citrate; (2) correlation between thalamic volume reduction and cerebral brain metric measures, both of which was similar to the term CHD group, suggesting concordance of pathogenesis of dysmaturation in the preterm control group and the term CHD group.

The reduced cerebellar volume (hypoplasia) in the preterm CHD group, we hypothesize, may be related to unknown genetic/intrinsic factors, as there would be relatively less exposure to in-utero substrate delivery compared to the term CHD and relatively increased energy demand because of the stress of the extra-uterine environment. Importantly, we have observed cerebellar hypoplasia and dysplasia in our preclinical models of CHD^42^. Of note, there was large variation in the preterm CHD cerebellar volumes compared to the other groups, and in the absence of cerebellar parenchymal injury, we hypothesize that this could reflect more subtle dysplasia (disproportion width and height of the cerebellar hemispheres and vermis) which we have recently described in term neonates CHD^13,43^. We did note fairly consistent results suggesting that reduced cerebellar volume is associated with reduced glutamine metabolism in the preterm and term CHD groups, but not in the control preterm group, suggesting that prematurity alone is not a driving factor. We also noted that single ventricle, aortic arch obstruction and cyanotic heart lesion diagnoses were predictive of reduced GLX (particularly glutamine metabolism). Importantly, we also noted that single ventricle, aortic arch obstruction and cyanotic lesion were predictive of the relationship of reduced subcortical morphology metrics (thalamus/cerebellum) and reduced GLX (mostly glutamine) in both CHD cohorts in frontal white matter/parietal grey matter. Taken together, we hypothesize that alterations in glutamine, an important metabolite related to TCA cycle metabolism and oxygen conformance, may underlie regional alterations in cellular energy metabolism, either due to substrate delivery reduction and/or intrinsic metabolic derangement (including oxygen conformance from chronic low hypoxia as proposed by Seed *et al*.^41^). We speculate that we might see this relationship with reduced cerebellar morphology and glutamine metabolism stronger in the preterm CHD group compared to the term CHD group, as while the preterm CHD group would be relatively less exposed to reduced fetal substrate delivery compared to term CHD infants, the preterm CHD infant would at risk for increased energy demand with earlier extra-uterine exposure, during a time that the cerebellum and subplate of the white matter is rapidly growing (critical period). The term CHD cerebellum may also be vulnerable, as there is a protracted period of development of the cerebellum that extends into the early postnatal period. The differential findings in relation to the preterm control cerebellum is intriguing as acquired injury of the cerebellum (i.e. hemorrhages) are relatively rare in CHD compared to preterms without CHD.

We also delineated important relationships between the subcortical morphology and neurotransmitter metabolites including glutamate. Glutamate the most abundant excitatory neurotransmitter and is essential to brain function. Glutamate can be converted into glutamine and vice versa within astrocytes and there exists a balance between glutamatergic and glutaminergic biochemical pathways^44^.

Compared to the term CHD group, the preterm CHD group demonstrated increased transverse cerebellar diameter with increased volume of CSF. We have recently described the presence of increased CSF volume and cerebellar dysplasia (also characterized by disproportionate size of the cerebellum relative to supratentorial structures) in a cohort of term CHD and it is possible that preterm CHD may also exhibit this dysplasia^13^. In future work, we will correlate our findings of brain dysplasia with brain metabolism. Some of these defects could also be related with intrinsic brain energy metabolism deficits, and not only secondary to deficient substrate delivery and resulting white matter injury.

## Limitations

Our study does have some important limitations. The preterm and term CHD cohorts that we studied were fairly heterogenous related to heart lesion subtypes and we had limited access to prenatal and perinatal records for better clinical characterization of our cohorts. Except for the manual segmentation of the volume of the thalamus, the cerebellar measurements and brain metrics were linear measurements. Even though our brain metrics methodology has been well validated in neonatal population^8,27–32^, more detailed volumetric segmentations may have yielded more accurate results. Our cohort also had few CHD patients that had both pre-operative and post-operative brain MRIs, compared to our recently published cohort^19^. Our dataset was also missing data-points as all three MRS voxel were not obtained on every patients. This was a dataset of clinically indicated MRI scans and the degree of white matter injury was higher than that seen in our research-obtained studies^19^. For the main multi-variate regression model, we *a priori* chose these three co-variates (PMA, timing of MRI relative to surgery, and WMI) as the literature and our own work has shown consistently that these variables can influence both morphological and metabolism measurements in the developing brain (both in prematurity and congenital heart disease). However, despite no difference in some demographic measures (i.e. gender) between the groups, we are not able to show that other important demographics and clinical risk predictors including prenatal variables (head circumference), and perinatal variables (Apgar scores) are matched in these cohort and may be contributing to variance. We also dichotomized our punctate white matter co-variate measure and did not account for a severity range or volume of PMWI in our multivariate regression model.

## Conclusion

Here, we delineate *in vivo* associations between subcortical (thalamic and cerebellar) morphological reductions and altered cerebral white matter metabolism in both preterm and term CHD infants. We did not find that these subcortical morphological reductions were associated with global alterations in brain maturation metabolites (NAA, choline and myo-inositol). However, instead, we noted selective associations between reduced subcortical volume and altered white matter energy metabolism which was not necessarily dependent upon prematurity or presence of white matter injury. Our findings are not representative of systemic energy failure, but we raise awareness that regional cerebral energy metabolism alterations do relate to reduced subcortical morphometry in CHD infants, extending our scope of metabolite alterations to include citrate and glutamine (beyond that of previously reported lactate and NAA only). This is supported by a convergence of findings related to: (1) reduced thalamic volume associated with citrate elevation in all three cohorts in the parietal white matter; (2) single ventricle, aortic arch obstruction and cyanotic heart lesion classification predicted the relationship between reduced cerebellar volume and reduced glutamine in the CHD cohorts localized frontal white matter/parietal grey matter. Our findings are hypothesis generating and need replication in separate cohorts and validation in preclinical CHD models for better mechanistic delineation. Taken together, these findings suggest that subplate vulnerability in CHD is likely relevant to understanding the mechanism of both cortical and subcortical dysmaturation in CHD infants. Future work is needed to link this potential pattern of encephalopathy of CHD (including the constellation of grey matter, white matter and brain metabolism deficits) to not only abnormal fetal substrate delivery and oxygen conformance, but also regional deficits in cerebral energy metabolism.

## Electronic supplementary material

Supplemental Tables S1-S9
