# Supplementary material for: Association between Subcortical Morphology and Cerebral White Matter Energy Metabolism in Neonates with Congenital Heart Disease
Source: Sci Rep. 2018 Sep 19;8:14057. doi: 10.1038/s41598-018-32288-3 (PMC6145929; doi:10.1038/s41598-018-32288-3)
Supplement: Supplementary file 1 — Supplemental Tables S1-S9 [file 41598_2018_32288_MOESM1_ESM.docx]

**Association between Subcortical Morphology and Cerebral White Matter Energy Metabolism in Neonates with Congenital Heart Disease**

Nina Gertsvolf, BS*;^1^ Jodie K. Votava-Smith, MD*;^1,2^ Rafael Ceschin, PhD;^3,4^

Sylvia del Castillo, MD; ^1,5^ Vince Lee, BS;^3^

Hollie A Lai ,MD ; ^1,6^ Stefan Bluml, PhD; ^1,6^

Lisa Paquette, MD**; ^1,7^ and Ashok Panigrahy, MD**; ^3,4,6^

1. Keck School of Medicine, University of Southern California, Los Angeles, CA
2. Department of Pediatrics, Division of Cardiology, Children’s Hospital of Los Angeles, Los Angeles, CA
3. Department of Pediatric Radiology, Children’s Hospital of Pittsburgh of UPMC and University of Pittsburgh School of Medicine
4. Department of Biomedical Informatics, University of Pittsburgh School of Medicine
5. Department of Anesthesiology, Critical Care Medicine Children’s Hospital of Los Angeles, Los Angeles, CA
6. Department of Radiology, Children’s Hospital of Los Angeles, Los Angeles, CA
7. Department of Pediatrics, Division of Neonatology, Children’s Hospital of Los Angeles, Los Angeles, CA

*co-first authors ; **co-senior authors

Table S1: Association between Cyanotic Heart Lesion Type and Parietal White Matter Metabolite Levels

| Metabolite | Term CHD |  |  | Cyanotic vs Non-Cyanotic | | Preterm CHD | |  | Cyanotic vs Non-Cyanotic | |
| --- | --- | --- | --- | --- | --- | --- | --- | --- | --- | --- |
|  | N | R^2^ | Coeff | p-value | direction | N | R2 | Coeff | p-value | direction |
| Cr | 47 | 0.184 | 18.133 | 0.3888 | *N.S.* | 33 | 0.667 | 13.531 | 0.3398 | *N.S.* |
| Gln | 48 | 0.001 | 53.592 | 0.8593 | *N.S.* | 33 | 0.034 | 36.079 | 0.4755 | *N.S.* |
| Glu | 48 | 0.217 | 41.675 | 0.1106 | *N.S.* | 33 | 0.534 | 31.269 | 0.8884 | *N.S.* |
| Glx | 48 | 0.073 | 36.068 | 0.4421 | *N.S.* | 33 | 0.248 | 25.357 | 0.5499 | *N.S.* |
| Ins | 48 | 0.072 | 20.161 | 0.7823 | *N.S.* | 33 | 0.092 | 23.293 | 0.5355 | *N.S.* |
| Lac | 48 | 0.059 | 169.546 | 0.1541 | *N.S.* | 33 | 0.086 | 95.696 | 0.8656 | *N.S.* |
| NAA | 48 | 0.4 | 32.267 | 0.7732 | *N.S.* | 33 | 0.872 | 19.958 | 0.4019 | *N.S.* |
| Cho | 48 | 0.032 | 22.239 | 0.9678 | *N.S.* | 33 | 0.061 | 15.894 | 0.3149 | *N.S.* |
| Cit | 48 | 0.191 | 42.137 | 0.3007 | *N.S.* | 33 | 0.495 | 36.364 | 0.2285 | *N.S.* |

Significant p-value bolded

Table S2: Association between Cyanotic Heart Lesion Type and Frontal White Matter Metabolite Levels

| Metabolite | Term CHD |  |  | Cyanotic vs Non-Cyanotic | | Preterm CHD | |  | Cyanotic vs Non-Cyanotic | |
| --- | --- | --- | --- | --- | --- | --- | --- | --- | --- | --- |
|  | N | R^2^ | Coeff | p-value | direction | N | R2 | Coeff | p-value | direction |
| Cr | 27 | 0.327 | 24.652 | 0.9467 | *N.S.* | 24 | 0.618 | 15.39 | 0.2711 | *N.S.* |
| Gln | 26 | 0.076 | 38.966 | 0.184 | *N.S.* | 24 | 0.133 | 36.757 | 0.1068 | *N.S.* |
| Glu | 26 | 0.477 | 33.114 | 0.3034 | *N.S.* | 24 | 0.398 | 34.069 | 0.2425 | *N.S.* |
| Glx | 26 | 0.202 | 31.195 | 0.7522 | *N.S.* | 24 | 0.465 | 16.697 | **0.0057** | **DEC.** |
| Ins | 26 | 0.021 | 23.516 | 0.4919 | *N.S.* | 24 | 0.214 | 17.564 | 0.6068 | *N.S.* |
| Lac | 26 | 0.216 | 71.951 | 0.5215 | *N.S.* | 24 | 0.205 | 85.975 | 0.7694 | *N.S.* |
| NAA | 26 | 0.705 | 19.94 | 0.5653 | *N.S.* | 24 | 0.771 | 23.235 | 0.4081 | *N.S.* |
| Cho | 26 | 0.159 | 21.547 | 0.8051 | *N.S.* | 24 | 0.061 | 19.139 | 0.3484 | *N.S.* |
| Cit | 26 | 0.265 | 48.052 | 0.3762 | *N.S.* | 24 | 0.289 | 48.977 | 0.2982 | *N.S.* |

Significant p-value bolded

Table S3: Association between Cyanotic Heart Lesion Type and Grey Matter Metabolite Levels

| Metabolite | Term CHD |  |  | Cyanotic vs Non-Cyanotic | | Preterm CHD | |  | Cyanotic vs Non-Cyanotic | |
| --- | --- | --- | --- | --- | --- | --- | --- | --- | --- | --- |
|  | N | R^2^ | Coeff | p-value | direction | N | R2 | Coeff | p-value | direction |
| Cr | 43 | 0.147 | 12.857 | 0.4948 | *N.S.* | 33 | 0.278 | 19.119 | 0.6241 | *N.S.* |
| Gln | 43 | 0.044 | 82.221 | 0.4154 | *N.S.* | 33 | 0.141 | 51.261 | **0.035** | **DEC.** |
| Glu | 43 | 0.241 | 45.546 | 0.2969 | *N.S.* | 33 | 0.372 | 40.681 | 0.503 | *N.S.* |
| Glx | 43 | 0.105 | 56.532 | 0.8649 | *N.S.* | 33 | 0.21 | 39.822 | 0.1154 | *N.S.* |
| Ins | 43 | 0.251 | 21.426 | 0.0876 | *N.S.* | 33 | 0.503 | 17.603 | 0.8207 | *N.S.* |
| Lac | 43 | 0 | 97.858 | 0.9439 | *N.S.* | 33 | 0.107 | 121.336 | 0.0682 | *N.S.* |
| NAA | 43 | 0.462 | 25.871 | 0.8249 | *N.S.* | 33 | 0.765 | 24.619 | 0.723 | *N.S.* |
| Cho | 43 | 0.039 | 23.207 | 0.3611 | *N.S.* | 33 | 0.031 | 21.36 | 0.5935 | *N.S.* |
| Cit | 43 | 0.147 | 47.682 | 0.7741 | *N.S.* | 33 | 0.398 | 51.478 | 0.9294 | *N.S.* |

Significant p-value bolded

Table S4: Association between Aortic Arch Obstruction and Parietal White Matter Metabolite Levels

| Metabolite | Term CHD |  |  | Obstructive vs. Non-obstructive | | Preterm CHD | |  | Obstructive vs. Non-obstructive | |
| --- | --- | --- | --- | --- | --- | --- | --- | --- | --- | --- |
|  | N | R^2^ | Coeff | p-value | direction | N | R2 | Coeff | p-value | direction |
| Cr | 46 | 0.21 | 17.144 | 0.319 | *N.S.* | 29 | 0.637 | 14.458 | 0.7131 | *N.S.* |
| Gln | 47 | 0.057 | 52.457 | 0.111 | *N.S.* | 29 | 0.003 | 33.459 | 0.7888 | *N.S.* |
| Glu | 47 | 0.27 | 41.002 | 0.0613 | *N.S.* | 29 | 0.517 | 31.737 | 0.6757 | *N.S.* |
| Glx | 47 | 0.162 | 35.525 | **0.0263** | **DEC.** | 29 | 0.29 | 25.138 | 0.6571 | *N.S.* |
| Ins | 47 | 0.09 | 18.825 | 0.9298 | *N.S.* | 29 | 0.137 | 21.363 | 0.815 | *N.S.* |
| Lac | 47 | 0.032 | 178.666 | 0.4126 | *N.S.* | 29 | 0.132 | 85.247 | 0.4203 | *N.S.* |
| NAA | 47 | 0.427 | 30.859 | 0.5291 | *N.S.* | 29 | 0.86 | 21.282 | 0.9248 | *N.S.* |
| Cho | 47 | 0.111 | 21.27 | 0.0612 | *N.S.* | 29 | 0.068 | 16.334 | 0.198 | *N.S.* |
| Cit | 47 | 0.192 | 43.089 | 0.6012 | *N.S.* | 29 | 0.609 | 33.197 | 0.0697 | *N.S.* |

Significant p-value bolded

Table S5: Association between Aortic Arch Obstruction and Frontal White Matter Metabolite Levels

| Metabolite | Term CHD |  |  | Obstructive vs. Non-obstructive | | Preterm CHD | |  | Obstructive vs. Non-obstructive | |
| --- | --- | --- | --- | --- | --- | --- | --- | --- | --- | --- |
|  | N | R^2^ | Coeff | p-value | direction | N | R2 | Coeff | p-value | direction |
| Cr | 28 | 0.333 | 24.025 | 0.7295 | *N.S.* | 21 | 0.62 | 16.057 | 0.7966 | *N.S.* |
| Gln | 27 | 0.046 | 38.677 | 0.2942 | *N.S.* | 21 | 0.004 | 29.7 | 0.9573 | *N.S.* |
| Glu | 27 | 0.526 | 30.925 | 0.079 | *N.S.* | 21 | 0.469 | 32.492 | 0.2786 | *N.S.* |
| Glx | 27 | 0.293 | 28.789 | 0.1013 | *N.S.* | 21 | 0.504 | 16.362 | 0.255 | *N.S.* |
| Ins | 27 | 0.017 | 23.11 | 0.5324 | *N.S.* | 21 | 0.132 | 18.043 | 0.9304 | *N.S.* |
| Lac | 27 | 0.218 | 73.466 | 0.6483 | *N.S.* | 21 | 0.286 | 78.873 | 0.9215 | *N.S.* |
| NAA | 27 | 0.705 | 19.605 | 0.9689 | *N.S.* | 21 | 0.793 | 23.49 | 0.9872 | *N.S.* |
| Cho | 27 | 0.159 | 21.08 | 0.8262 | *N.S.* | 21 | 0.056 | 19.715 | 0.7157 | *N.S.* |
| Cit | 27 | 0.254 | 47.785 | 0.5426 | *N.S.* | 21 | 0.442 | 36.929 | 0.3963 | *N.S.* |

Significant p-value bolded

Table S6: Association between Aortic Arch Obstruction and Grey Matter Metabolite Levels

| Metabolite | Term CHD |  |  | Obstructive vs. Non-obstructive | | Preterm CHD | |  | Obstructive vs. Non-obstructive | |
| --- | --- | --- | --- | --- | --- | --- | --- | --- | --- | --- |
|  | N | R^2^ | Coeff | p-value | direction | N | R2 | Coeff | p-value | direction |
| Cr | 43 | 0.131 | 13 | 0.871 | *N.S.* | 29 | 0.274 | 20.35 | 0.9263 | *N.S.* |
| Gln | 43 | 0.088 | 80.308 | 0.1034 | *N.S.* | 29 | 0.036 | 54.842 | 0.4273 | *N.S.* |
| Glu | 43 | 0.376 | 40.86 | **0.015** | **DEC.** | 29 | 0.373 | 41.647 | 0.7874 | *N.S.* |
| Glx | 43 | 0.211 | 53.248 | **0.0313** | **DEC.** | 29 | 0.211 | 41.695 | 0.549 | *N.S.* |
| Ins | 43 | 0.2 | 22.112 | 0.7485 | *N.S.* | 29 | 0.457 | 18.575 | 0.6091 | *N.S.* |
| Lac | 43 | 0.026 | 99.588 | 0.32 | *N.S.* | 29 | 0.001 | 125.922 | 0.851 | *N.S.* |
| NAA | 43 | 0.52 | 24.441 | 0.0689 | *N.S.* | 29 | 0.768 | 25.332 | 0.3743 | *N.S.* |
| Cho | 43 | 0.063 | 22.92 | 0.1853 | *N.S.* | 29 | 0.031 | 22.849 | 0.4829 | *N.S.* |
| Cit | 43 | 0.139 | 48.187 | 0.7368 | *N.S.* | 29 | 0.519 | 47.465 | 0.2672 | *N.S.* |

Significant p-value bolded

Table S7: Association between Single-Ventricle Heart Lesion and Parietal White Matter Metabolite Levels

| Metabolite | Term CHD |  |  | Double vs. Single | | Preterm CHD | |  | Double vs. Single | |
| --- | --- | --- | --- | --- | --- | --- | --- | --- | --- | --- |
|  | N | R^2^ | Coeff | p-value | direction | N | R2 | Coeff | p-value | direction |
| Cr | 46 | 0.191 | 17.342 | 0.8918 | *N.S.* | 29 | 0.641 | 14.375 | 0.512 | *N.S.* |
| Gln | 47 | 0.02 | 53.486 | 0.3566 | *N.S.* | 29 | 0.01 | 33.347 | 0.622 | *N.S.* |
| Glu | 47 | 0.239 | 41.852 | 0.1901 | *N.S.* | 29 | 0.527 | 31.387 | 0.3893 | *N.S.* |
| Glx | 47 | 0.103 | 36.758 | 0.1609 | *N.S.* | 29 | 0.305 | 24.862 | 0.3832 | *N.S.* |
| Ins | 47 | 0.09 | 18.827 | 0.9724 | *N.S.* | 29 | 0.136 | 21.368 | 0.8355 | *N.S.* |
| Lac | 47 | 0.023 | 179.488 | 0.6021 | *N.S.* | 29 | 0.111 | 86.289 | 0.8628 | *N.S.* |
| NAA | 47 | 0.427 | 30.859 | 0.529 | *N.S.* | 29 | 0.86 | 21.274 | 0.8657 | *N.S.* |
| Cho | 47 | 0.043 | 22.07 | 0.5897 | *N.S.* | 29 | 0.007 | 16.857 | 0.8232 | *N.S.* |
| Cit | 47 | 0.187 | 43.222 | 0.9381 | *N.S.* | 29 | 0.571 | 34.772 | 0.3362 | *N.S.* |

Significant p-value bolded

Table S8: Association between Single Ventricle Heart Lesion and Frontal White Matter Metabolite Levels

| Metabolite | Term CHD |  |  | Double vs. Single | | Preterm CHD | |  | Double vs. Single | |
| --- | --- | --- | --- | --- | --- | --- | --- | --- | --- | --- |
|  | N | R^2^ | Coeff | p-value | direction | N | R2 | Coeff | p-value | direction |
| Cr | 28 | 0.339 | 23.904 | 0.5448 | *N.S.* | 21 | 0.658 | 15.228 | 0.1657 | *N.S.* |
| Gln | 27 | 0.051 | 38.584 | 0.2707 | *N.S.* | 21 | 0.009 | 29.635 | 0.778 | *N.S.* |
| Glu | 27 | 0.482 | 32.327 | 0.3173 | *N.S.* | 21 | 0.545 | 30.08 | **0.049** | **INC.** |
| Glx | 27 | 0.208 | 30.459 | 0.8564 | *N.S.* | 21 | 0.603 | 14.635 | **0.0225** | **INC.** |
| Ins | 27 | 0.008 | 23.207 | 0.6604 | *N.S.* | 21 | 0.224 | 17.061 | 0.1608 | *N.S.* |
| Lac | 27 | 0.32 | 68.531 | 0.0622 | *N.S.* | 21 | 0.286 | 78.89 | 0.9638 | *N.S.* |
| NAA | 27 | 0.725 | 18.938 | 0.2019 | *N.S.* | 21 | 0.804 | 22.886 | 0.3395 | *N.S.* |
| Cho | 27 | 0.189 | 20.69 | 0.3356 | *N.S.* | 21 | 0.12 | 19.033 | 0.2428 | *N.S.* |
| Cit | 27 | 0.335 | 45.109 | 0.0792 | *N.S.* | 21 | 0.418 | 37.693 | 0.9561 | *N.S.* |

Significant p-value bolded

Table S9: Association between Single Ventricle Heart Lesion and Grey Matter Metabolite Levels

| Metabolite | Term CHD |  |  | Double vs. Single | | Preterm CHD | |  | Double vs. Single | |
| --- | --- | --- | --- | --- | --- | --- | --- | --- | --- | --- |
|  | N | R^2^ | Coeff | p-value | direction | N | R2 | Coeff | p-value | direction |
| Cr | 43 | 0.162 | 12.77 | 0.2301 | *N.S.* | 29 | 0.282 | 20.237 | 0.5884 | *N.S.* |
| Gln | 43 | 0.025 | 83.041 | 0.9294 | *N.S.* | 29 | 0.025 | 55.147 | 0.5558 | *N.S.* |
| Glu | 43 | 0.312 | 42.903 | 0.1513 | *N.S.* | 29 | 0.391 | 41.069 | 0.3753 | *N.S.* |
| Glx | 43 | 0.118 | 56.298 | 0.6274 | *N.S.* | 29 | 0.222 | 41.417 | 0.4024 | *N.S.* |
| Ins | 43 | 0.203 | 22.065 | 0.6019 | *N.S.* | 29 | 0.464 | 18.465 | 0.4524 | *N.S.* |
| Lac | 43 | 0.012 | 100.308 | 0.5171 | *N.S.* | 29 | 0.005 | 125.697 | 0.7221 | *N.S.* |
| NAA | 43 | 0.501 | 24.915 | 0.1808 | *N.S.* | 29 | 0.765 | 25.462 | 0.4671 | *N.S.* |
| Cho | 43 | 0.023 | 23.409 | 0.7691 | *N.S.* | 29 | 0.025 | 22.926 | 0.5717 | *N.S.* |
| Cit | 43 | 0.137 | 48.254 | 0.9457 | *N.S.* | 29 | 0.497 | 48.547 | 0.7752 | *N.S.* |

Significant p-value bolded

| Table S10: Parietal White Matter Metabolite vs Transverse Cerebellar Distance (Cyanotic Lesions) | | | | | | |
| --- | --- | --- | --- | --- | --- | --- |
|  |  |  |  |  |  |  |
| Metabolite | Term CHD | | | Preterm CHD | | |
|  | R^2^ | Coeff | p-value of lesion | R^2^ | Coeff | p-value of lesion |
| Cr | 0.192631 | 18.24588 | 0.375 | 0.686974 | 13.3466 | 0.2589 |
| Gln | 0.013443 | 54.00253 | 0.9476 | 0.035321 | 36.6718 | 0.5023 |
| Glu | 0.26531 | 38.79053 | **0.0418** | 0.564492 | 30.73517 | 0.7442 |
| Glx | 0.101495 | 35.03632 | 0.2906 | 0.261628 | 25.56231 | 0.4965 |
| Ins | 0.077575 | 19.22979 | 0.9576 | 0.143546 | 23.00928 | 0.4295 |
| Lac | 0.155852 | 90.45774 | 0.1828 | 0.148034 | 93.9843 | 0.9869 |
| NAA | 0.439631 | 30.93571 | 0.9384 | 0.912746 | 16.77492 | 0.1493 |
| Cho | 0.172871 | 15.35544 | 0.2682 | 0.071049 | 16.07633 | 0.2907 |
| Cit | 0.230707 | 39.62167 | 0.4767 | 0.532679 | 35.56855 | 0.3042 |
| Term CHD N = 47 |  |  |  |  |  |  |
| Preterm CHD N = 33  Significant p-value bolded |  |  |  |  |  |  |

| Table S11: Frontal White Matter Metabolite vs Transverse Cerebellar Distance (Cyanotic Lesion) | | | | | | |
| --- | --- | --- | --- | --- | --- | --- |
|  |  |  |  |  |  |  |
| Metabolite | Term CHD |  |  | Preterm CHD |  |  |
|  | R^2^ | Coeff | p-value of lesion | R^2^ | Coeff | p-value of lesion |
| Cr | 0.497396 | 19.25109 | 0.7217 | 0.663654 | 14.7962 | 0.1639 |
| Gln | 0.095917 | 39.40543 | 0.1614 | 0.135253 | 37.61997 | 0.1291 |
| Glu | 0.480421 | 33.74763 | 0.2916 | 0.474143 | 32.61768 | 0.1406 |
| Glx | 0.204396 | 31.85455 | 0.7316 | 0.517824 | 16.23701 | **0.003** |
| Ins | 0.236769 | 21.2278 | 0.2571 | 0.230516 | 17.80844 | 0.5414 |
| Lac | 0.218048 | 73.47348 | 0.5114 | 0.433662 | 74.37571 | 0.4145 |
| NAA | 0.712297 | 20.1513 | 0.5002 | 0.838511 | 20.0073 | 0.1575 |
| Cho | 0.295465 | 20.16226 | 0.5583 | 0.122359 | 18.96318 | 0.2601 |
| Cit | 0.265704 | 49.10857 | 0.3806 | 0.386869 | 46.59098 | 0.172 |
| Term CHD N = 26 |  |  |  |  |  |  |
| Preterm CHD N = 24 |  |  |  |  |  |  |

Significant p-value bolded

| Table S12: Grey Matter Metabolite vs Transverse Cerebellar Distance ( Cyanotic Lesions) | | | | | | |
| --- | --- | --- | --- | --- | --- | --- |
|  |  |  |  |  |  |  |
| Metabolite | Term CHD |  |  | Preterm CHD |  |  |
|  | R^2^ | Coeff | p-value of lesion | R^2^ | Coeff | p-value of lesion |
| Cr | 0.183206 | 12.73993 | 0.4688 | 0.294103 | 19.22788 | 0.7127 |
| Gln | 0.141746 | 78.89274 | 0.3653 | 0.32089 | 46.34638 | 0.0522 |
| Glu | 0.240944 | 46.12607 | 0.3032 | 0.427109 | 39.52089 | 0.6532 |
| Glx | 0.152741 | 55.69128 | 0.8308 | 0.353877 | 36.63421 | 0.1768 |
| Ins | 0.258619 | 21.594 | 0.0873 | 0.519921 | 17.60406 | 0.9331 |
| Lac | 0.026041 | 97.82393 | 0.9217 | 0.12517 | 122.1206 | 0.0908 |
| NAA | 0.489659 | 25.50704 | 0.7913 | 0.77249 | 24.64561 | 0.6272 |
| Cho | 0.045592 | 23.42289 | 0.3584 | 0.116686 | 20.74676 | 0.7568 |
| Cit | 0.148896 | 48.24208 | 0.7709 | 0.397977 | 52.34433 | 0.945 |
| Term CHD N = 43 |  |  |  |  |  |  |
| Preterm CHD N = 33  Significant p-value bolded |  |  |  |  |  |  |

| Table S13: Parietal White Matter Metabolite vs Transverse Cerebellar Distance (Arch Obstruction) | | | | | | |
| --- | --- | --- | --- | --- | --- | --- |
| Metabolite | Term CHD |  |  | Preterm CHD |  |  |
|  | R^2^ | Coeff | p-value of lesion | R^2^ | Coeff | p-value of lesion |
| Cr | 0.210839 | 17.33384 | 0.3516 | 0.647909 | 14.52228 | 0.8463 |
| Gln | 0.061121 | 53.11563 | 0.1668 | 0.024006 | 33.76368 | 0.6907 |
| Glu | 0.269916 | 39.43599 | 0.1249 | 0.562246 | 30.8067 | 0.9048 |
| Glx | 0.150865 | 35.35103 | 0.0591 | 0.296117 | 25.52046 | 0.734 |
| Ins | 0.11065 | 17.60675 | 0.5725 | 0.171887 | 21.33888 | 0.6719 |
| Lac | 0.134476 | 96.26086 | 0.5704 | 0.190771 | 83.96155 | 0.2939 |
| NAA | 0.484173 | 29.04198 | 0.23 | 0.897439 | 18.58986 | 0.6444 |
| Cho | 0.173153 | 15.15707 | 0.2674 | 0.092412 | 16.43502 | 0.1605 |
| Cit | 0.244832 | 40.09713 | 0.8888 | 0.700297 | 29.65216 | **0.0153** |
| Term CHD N = 46 | |  |  |  |  |  |
| Preterm CHD N = 29 | |  |  |  |  |  |

Significant p-value bolded

| Table S14: Frontal White Matter Metabolite vs Transverse Cerebellar Distance (Arch Obstruction) | | | | | | |
| --- | --- | --- | --- | --- | --- | --- |
| Metabolite | Term CHD |  |  | Preterm CHD |  |  |
|  | R^2^ | Coeff | p-value of lesion | R^2^ | Coeff | p-value of lesion |
| Cr | 0.50054 | 18.74734 | 0.5387 | 0.644862 | 15.96477 | 0.6921 |
| Gln | 0.055079 | 39.3254 | 0.3072 | 0.04161 | 29.98264 | 0.876 |
| Glu | 0.52658 | 31.56696 | 0.0849 | 0.489947 | 32.77543 | 0.3356 |
| Glx | 0.293911 | 29.37902 | 0.1096 | 0.504251 | 16.83084 | 0.2786 |
| Ins | 0.214873 | 21.0944 | 0.4623 | 0.133433 | 18.55173 | 0.9494 |
| Lac | 0.218547 | 75.03696 | 0.6542 | 0.456494 | 70.80845 | 0.688 |
| NAA | 0.71062 | 19.82693 | 0.9813 | 0.833817 | 21.66501 | 0.7823 |
| Cho | 0.284054 | 19.86237 | 0.7817 | 0.076399 | 20.06282 | 0.6654 |
| Cit | 0.25366 | 48.81267 | 0.5512 | 0.498158 | 36.03042 | 0.3033 |
| term CHD N = 27 | |  |  |  |  |  |
| Preterm CHD N= 21 | |  |  |  |  |  |

Significant p-value bolded

| Table S15: Parietal Grey Matter Metabolite vs Transverse Cerebellar Distance (Arch Obstruction) | | | | | | |
| --- | --- | --- | --- | --- | --- | --- |
| Metabolite | Term CHD |  |  | Preterm CHD |  |  |
|  | R^2^ | Coeff | p-value of lesion | R^2^ | Coeff | p-value of lesion |
| Cr | 0.167219 | 12.89078 | 0.9438 | 0.290541 | 20.50976 | 0.9864 |
| Gln | 0.173524 | 77.43267 | 0.1224 | 0.296931 | 47.76913 | 0.1904 |
| Glu | 0.376961 | 41.33733 | **0.0157** | 0.435546 | 40.30739 | 0.6125 |
| Glx | 0.24752 | 52.67359 | **0.0377** | 0.387185 | 37.48447 | 0.3054 |
| Ins | 0.207653 | 22.2849 | 0.7178 | 0.476755 | 18.59823 | 0.7105 |
| Lac | 0.047574 | 99.73414 | 0.3555 | 0.03836 | 126.0203 | 0.9609 |
| NAA | 0.556041 | 23.79645 | **0.0478** | 0.771721 | 25.61839 | 0.4351 |
| Cho | 0.066626 | 23.16793 | 0.2005 | 0.112196 | 22.30645 | 0.6153 |
| Cit | 0.140581 | 48.76038 | 0.7267 | 0.522266 | 48.24969 | 0.2566 |
| Term CHD N=43 | |  |  |  |  |  |
| Preterm CHD N=29 | |  |  |  |  |  |

Significant p-value bolded

| Table S16: Parietal White Matter Metabolite vs Transverse Cerebellar Distance (Single Ventricle) | | | | | | |
| --- | --- | --- | --- | --- | --- | --- |
| Metabolite | Term CHD |  |  | Preterm CHD |  |  |
|  | R^2^ | Coeff | p-value of lesion | R^2^ | Coeff | p-value of lesion |
| Cr | 0.194449 | 17.51292 | 0.9038 | 0.655306 | 14.36894 | 0.4551 |
| Gln | 0.0336 | 53.88846 | 0.3987 | 0.024937 | 33.74758 | 0.6699 |
| Glu | 0.252361 | 39.90731 | 0.242 | 0.581512 | 30.12114 | 0.2905 |
| Glx | 0.110063 | 36.19041 | 0.204 | 0.316671 | 25.14507 | 0.3589 |
| Ins | 0.104424 | 17.66827 | 0.8649 | 0.16886 | 21.37784 | 0.7642 |
| Lac | 0.128001 | 96.62023 | 0.9101 | 0.153719 | 85.86216 | 0.9468 |
| NAA | 0.46857 | 29.47793 | 0.6516 | 0.897498 | 18.58454 | 0.6337 |
| Cho | 0.148865 | 15.37808 | 0.8666 | 0.017629 | 17.09873 | 0.865 |
| Cit | 0.244869 | 40.09616 | 0.8833 | 0.629439 | 32.97162 | 0.4092 |
| Term CHD N=46 | |  |  |  |  |  |
| Preterm CHD N=29 | |  |  |  |  |  |

Significant p-value bolded

| Table S17: Frontal White Matter Metabolite vs Transverse Cerebellar Distance (Single Ventricle ) | | | | | | |
| --- | --- | --- | --- | --- | --- | --- |
| Metabolite | Term CHD |  |  | Preterm CHD |  |  |
|  | R^2^ | Coeff | p-value of lesion | R^2^ | Coeff | p-value of lesion |
| Cr | 0.504103 | 18.68036 | 0.4628 | 0.711532 | 14.3884 | 0.0581 |
| Gln | 0.053338 | 39.36161 | 0.3171 | 0.040215 | 30.00445 | 0.9853 |
| Glu | 0.482089 | 33.01695 | 0.3305 | 0.632323 | 27.82749 | **0.0118** |
| Glx | 0.209438 | 31.08677 | 0.8978 | 0.634235 | 14.45693 | **0.0128** |
| Ins | 0.195852 | 21.34839 | 0.9674 | 0.245797 | 17.30722 | 0.1296 |
| Lac | 0.323779 | 69.80206 | 0.063 | 0.46889 | 69.99633 | 0.4615 |
| NAA | 0.726681 | 19.26884 | 0.2568 | 0.865005 | 19.52652 | 0.061 |
| Cho | 0.292618 | 19.74322 | 0.5555 | 0.174306 | 18.96964 | 0.1535 |
| Cit | 0.339715 | 45.9124 | 0.0777 | 0.469126 | 37.05796 | 0.7171 |
| Term CHD N = 27 | |  |  |  |  |  |
| Preterm CHD N = 21 | |  |  |  |  |  |

Significant p-value bolded

| Table S18: Grey Matter Metabolite vs Transverse Cerebellar Distance (Single Ventricle) | | | | | | |
| --- | --- | --- | --- | --- | --- | --- |
| Metabolite | Term CHD |  |  | Preterm CHD |  |  |
|  | R^2^ | Coeff | p-value of lesion | R^2^ | Coeff | p-value of lesion |
| Cr | 0.194374 | 12.67886 | 0.2576 | 0.295444 | 20.43877 | 0.6799 |
| Gln | 0.120694 | 79.86919 | 0.9741 | 0.285773 | 48.14668 | 0.249 |
| Glu | 0.311673 | 43.44926 | 0.1575 | 0.462403 | 39.33678 | 0.2283 |
| Glx | 0.165535 | 55.46887 | 0.5613 | 0.407636 | 36.85369 | 0.1701 |
| Ins | 0.209751 | 22.25539 | 0.6296 | 0.492438 | 18.31739 | 0.3472 |
| Lac | 0.038879 | 100.1883 | 0.4778 | 0.049206 | 125.3076 | 0.5964 |
| NAA | 0.528592 | 24.52105 | 0.2049 | 0.769407 | 25.74789 | 0.5473 |
| Cho | 0.028627 | 23.63484 | 0.7493 | 0.10676 | 22.37465 | 0.7483 |
| Cit | 0.137915 | 48.83597 | 0.9573 | 0.498655 | 49.42763 | 0.7461 |
| Term CHD N=43 | |  |  |  |  |  |
| Preterm CHD N=29 | |  |  |  |  |  |

Significant p-value bolded

| Table S19: Parietal White Matter Metabolite vs Thalamic Volume (Cyanotic ) | | | | | | |
| --- | --- | --- | --- | --- | --- | --- |
| Metabolite | Term CHD |  |  | Preterm CHD |  |  |
|  | R^2^ | Coeff | p-value of lesion | R^2^ | Coeff | p-value of lesion |
| Cr | 0.236336 | 17.74515 | 0.286 | 0.690867 | 13.26336 | 0.3847 |
| Gln | 0.002414 | 54.30355 | 0.892 | 0.11281 | 35.16812 | 0.5355 |
| Glu | 0.290696 | 38.11447 | **0.0293** | 0.53442 | 31.7786 | 0.9022 |
| Glx | 0.091185 | 35.23674 | 0.2908 | 0.287481 | 25.11081 | 0.6065 |
| Ins | 0.106251 | 18.92852 | 0.8459 | 0.09713 | 23.62456 | 0.5241 |
| Lac | 0.12207 | 92.25 | 0.2182 | 0.126839 | 95.14619 | 0.8023 |
| NAA | 0.483294 | 29.70605 | 0.9032 | 0.876044 | 19.99407 | 0.4414 |
| Cho | 0.092315 | 16.08582 | 0.3837 | 0.102063 | 15.80569 | 0.3528 |
| Cit | 0.269864 | 38.60013 | 0.6358 | 0.579146 | 33.75393 | 0.2587 |
| Term CHD N=47 |  |  |  |  |  |  |
| Preterm CHD N=33 |  |  |  |  |  |  |

Significant p-value bolded

| Table S20: Frontal White Matter Metabolite vs Thalamic Volume (Cyanotic) | | | | | | |
| --- | --- | --- | --- | --- | --- | --- |
| Metabolite | Term CHD | | | Preterm CHD | | |
|  | R^2^ | Coeff | p-value of lesion | R^2^ | Coeff | p-value of lesion |
| Cr | 0.442711 | 20.27134 | 0.7019 | 0.631485 | 15.4876 | 0.2456 |
| Gln | 0.121413 | 38.84583 | 0.1167 | 0.148646 | 37.32751 | 0.1258 |
| Glu | 0.477682 | 33.83646 | 0.3129 | 0.419418 | 34.27292 | 0.2191 |
| Glx | 0.214089 | 31.65991 | 0.6402 | 0.466072 | 17.08616 | **0.0068** |
| Ins | 0.051829 | 23.66031 | 0.3683 | 0.214644 | 17.99117 | 0.6243 |
| Lac | 0.219199 | 73.41938 | 0.4921 | 0.327317 | 81.0585 | 0.6323 |
| NAA | 0.70549 | 20.38829 | 0.5865 | 0.773347 | 23.7027 | 0.3987 |
| Cho | 0.245347 | 20.86708 | 0.4753 | 0.111612 | 19.07893 | 0.3042 |
| Cit | 0.28451 | 48.47565 | 0.5428 | 0.292227 | 50.05786 | 0.3244 |
| Term CHD N=26 |  |  |  |  |  |  |
| Preterm CHD N=24 |  |  |  |  |  |  |

Significant p-value bolded

| Table S21: Grey Matter Metabolite Levels vs Thalamic Volume (Cyanotic ) | | | | | | |
| --- | --- | --- | --- | --- | --- | --- |
| Metabolite | Term CHD |  |  | Preterm CHD |  |  |
|  | R^2^ | Coeff | p-value of lesion | R^2^ | Coeff | p-value of lesion |
| Cr | 0.147704 | 13.01386 | 0.845 | 0.279934 | 19.41991 | 0.7808 |
| Gln | 0.065264 | 82.33296 | 0.3509 | 0.176265 | 51.04338 | 0.2716 |
| Glu | 0.25848 | 45.59015 | 0.3428 | 0.373705 | 41.32191 | 0.7836 |
| Glx | 0.106602 | 57.18755 | 0.7689 | 0.215737 | 40.36085 | 0.6548 |
| Ins | 0.299909 | 20.98407 | 0.1081 | 0.508264 | 17.8165 | 0.5975 |
| Lac | 0.051783 | 96.52254 | 0.1539 | 0.124913 | 122.1386 | 0.4422 |
| NAA | 0.487441 | 25.56239 | 0.1681 | 0.768596 | 24.85563 | 0.5166 |
| Cho | 0.098648 | 22.76253 | 0.1164 | 0.031872 | 21.71997 | 0.9088 |
| Cit | 0.15675 | 48.01896 | 0.5107 | 0.397918 | 52.34685 | 0.9126 |
| Term CHD N=43 |  |  |  |  |  |  |
| Preterm CHD N=33 |  |  |  |  |  |  |

| Table S22: Parietal White Matter Metabolite vs Thalamic Volume (Aortic Arch Obstruction) | | | | | | |
| --- | --- | --- | --- | --- | --- | --- |
| Metabolite | Term CHD |  |  | Preterm CHD |  |  |
|  | R^2^ | Coeff | p-value of lesion | R^2^ | Coeff | p-value of lesion |
| Cr | 0.227378 | 17.15124 | 0.2882 | 0.664792 | 14.16982 | 0.9887 |
| Gln | 0.053518 | 53.33024 | 0.1334 | 0.073944 | 32.88856 | 0.9214 |
| Glu | 0.2771 | 39.2415 | 0.1141 | 0.518184 | 32.31994 | 0.7438 |
| Glx | 0.152401 | 35.31906 | **0.0451** | 0.321738 | 25.05168 | 0.8837 |
| Ins | 0.123901 | 17.47509 | 0.5904 | 0.173183 | 21.32217 | 0.9614 |
| Lac | 0.106657 | 97.79557 | 0.4433 | 0.198326 | 83.5687 | 0.2474 |
| NAA | 0.510499 | 28.29116 | 0.1723 | 0.865679 | 21.27443 | 0.8627 |
| Cho | 0.14729 | 15.3923 | 0.1947 | 0.145119 | 15.95067 | 0.1011 |
| Cit | 0.25666 | 39.78188 | 0.9526 | 0.650456 | 32.02295 | 0.1628 |
| Term CHD N=46 |  |  |  |  |  |  |
| Preterm CHD N=29 |  |  |  |  |  |  |

Significant p-value bolded

| Table S23: Frontal White Matter Metabolite vs Thalamic Volume (Aortic Arch Obstruction) | | | | | | |
| --- | --- | --- | --- | --- | --- | --- |
| Metabolite | Term CHD |  |  | Preterm CHD |  |  |
|  | R^2^ | Coeff | p-value of lesion | R^2^ | Coeff | p-value of lesion |
| Cr | 0.631982 | 16.2517 | 0.6558 | 0.446035 | 19.74379 | 0.5779 |
| Gln | 0.006458 | 30.52755 | 0.9187 | 0.060648 | 39.20935 | 0.3044 |
| Glu | 0.469449 | 33.42752 | 0.3008 | 0.526689 | 31.56332 | 0.0859 |
| Glx | 0.505794 | 16.80463 | 0.2577 | 0.300237 | 29.24711 | 0.1083 |
| Ins | 0.149623 | 18.37761 | 0.8099 | 0.034697 | 23.38994 | 0.5324 |
| Lac | 0.416551 | 73.36425 | 0.5354 | 0.218421 | 75.04302 | 0.655 |
| NAA | 0.793327 | 24.1606 | 0.9621 | 0.704875 | 20.02276 | 0.9686 |
| Cho | 0.080499 | 20.01823 | 0.6 | 0.228159 | 20.62314 | 0.809 |
| Cit | 0.453249 | 37.60803 | 0.5184 | 0.286008 | 47.74313 | 0.5506 |
| Term CHD N=27 |  |  |  |  |  |  |
| Preterm CHD N=21 |  |  |  |  |  |  |

Significant p-value bolded

| Table S24: Grey Matter Metabolite vs Thalamic Volume (Aortic Arch Obstruction) | | | | | | |
| --- | --- | --- | --- | --- | --- | --- |
| Metabolite | Term CHD |  |  | Preterm CHD |  |  |
|  | R^2^ | Coeff | p-value of lesion | R^2^ | Coeff | p-value of lesion |
| Cr | 0.13152 | 13.16417 | 0.8655 | 0.274012 | 20.74731 | 0.898 |
| Gln | 0.112259 | 80.25136 | 0.0858 | 0.093119 | 54.25295 | 0.2362 |
| Glu | 0.375693 | 41.37939 | **0.0167** | 0.374211 | 42.44087 | 0.8589 |
| Glx | 0.223821 | 53.4966 | **0.0275** | 0.221709 | 42.24328 | 0.4504 |
| Ins | 0.228147 | 21.99483 | 0.8407 | 0.458488 | 18.92008 | 0.7034 |
| Lac | 0.079239 | 98.06218 | 0.2496 | 0.030325 | 126.5457 | 0.6293 |
| NAA | 0.530135 | 24.4809 | 0.0858 | 0.780826 | 25.10227 | 0.2085 |
| Cho | 0.137991 | 22.26462 | 0.1254 | 0.031375 | 23.29968 | 0.5083 |
| Cit | 0.145966 | 48.6074 | 0.6986 | 0.524412 | 48.1412 | 0.2294 |
| Term CHD N=43 |  |  |  |  |  |  |
| Preterm CHD N=29 |  |  |  |  |  |  |

Significant p-value bolded

| Table S25: Parietal White Matter Metabolite vs Thalamic Volume (Single Ventricle) | | | | | | |
| --- | --- | --- | --- | --- | --- | --- |
| Metabolite | Term CHD |  |  | Preterm CHD |  |  |
|  | R^2^ | Coeff | p-value of lesion | R^2^ | Coeff | p-value of lesion |
| Cr | 0.207862 | 17.36651 | 0.7606 | 0.677521 | 13.8982 | 0.33 |
| Gln | 0.019691 | 54.27489 | 0.3728 | 0.096515 | 32.48529 | 0.4331 |
| Glu | 0.262888 | 39.62536 | 0.1937 | 0.532988 | 31.81954 | 0.3505 |
| Glx | 0.107777 | 36.23685 | 0.1698 | 0.355293 | 24.42414 | 0.2607 |
| Ins | 0.117776 | 17.53607 | 0.9802 | 0.173106 | 21.32317 | 0.9949 |
| Lac | 0.093969 | 98.48762 | 0.9608 | 0.153396 | 85.87856 | 0.9685 |
| NAA | 0.488525 | 28.91921 | 0.8403 | 0.866238 | 21.23011 | 0.7163 |
| Cho | 0.112058 | 15.70706 | 0.9725 | 0.046041 | 16.84965 | 0.9765 |
| Cit | 0.256672 | 39.78157 | 0.9485 | 0.653145 | 31.89955 | 0.1438 |
| Term CHD N=46 |  |  |  |  |  |  |
| Preterm CHD N=29 |  |  |  |  |  |  |

Significant p-value bolded

| Table S26: Frontal White Matter Metabolite vs Thalamic Volume (Single Ventricle) | | | | | | |
| --- | --- | --- | --- | --- | --- | --- |
| Metabolite | Term CHD |  |  | Preterm CHD |  |  |
|  | R^2^ | Coeff | p-value of lesion | R^2^ | Coeff | p-value of lesion |
| Cr | 0.439889 | 19.85301 | 0.8043 | 0.684774 | 15.04093 | 0.0969 |
| Gln | 0.070834 | 38.99618 | 0.2541 | 0.00911 | 30.48677 | 0.8153 |
| Glu | 0.482296 | 33.01035 | 0.3343 | 0.566385 | 30.21987 | **0.0359** |
| Glx | 0.216967 | 30.93837 | 0.8266 | 0.61957 | 14.74389 | **0.0179** |
| Ins | 0.028163 | 23.46897 | 0.6256 | 0.225007 | 17.54413 | 0.2072 |
| Lac | 0.320981 | 69.94632 | 0.0665 | 0.412464 | 73.6208 | 0.6041 |
| NAA | 0.724503 | 19.34549 | 0.213 | 0.805703 | 23.42604 | 0.3121 |
| Cho | 0.267326 | 20.09306 | 0.2673 | 0.16777 | 19.04458 | 0.1657 |
| Cit | 0.359438 | 45.22148 | 0.0943 | 0.442402 | 37.97926 | 0.7608 |
| Term CHD N=27 |  |  |  |  |  |  |
| Preterm CHD N=21 |  |  |  |  |  |  |

Significant p-value bolded

| Table S27: Grey Matter Metabolite vs Thalamic Volume (Single Ventricle ) | | | | | | |
| --- | --- | --- | --- | --- | --- | --- |
| Metabolite | Term CHD |  |  | Preterm CHD |  |  |
|  | R^2^ | Coeff | p-value of lesion | R_2_ | Coeff | p-value of lesion |
| Cr | 0.162334 | 12.92853 | 0.2335 | 0.281638 | 20.63805 | 0.5998 |
| Gln | 0.041973 | 83.36774 | 0.8929 | 0.048788 | 55.56316 | 0.6291 |
| Glu | 0.313033 | 43.40631 | 0.1525 | 0.394358 | 41.75211 | 0.3613 |
| Glx | 0.124085 | 56.82982 | 0.6514 | 0.223377 | 42.198 | 0.4302 |
| Ins | 0.234403 | 21.90551 | 0.552 | 0.465837 | 18.79127 | 0.4885 |
| Lac | 0.05523 | 99.33243 | 0.563 | 0.029037 | 126.6297 | 0.6544 |
| NAA | 0.518091 | 24.79266 | 0.1597 | 0.76995 | 25.71753 | 0.5299 |
| Cho | 0.084817 | 22.94106 | 0.8355 | 0.027574 | 23.34535 | 0.5589 |
| Cit | 0.142666 | 48.70122 | 0.9694 | 0.497053 | 49.50653 | 0.7856 |
| Term CHD N=43 |  |  |  |  |  |  |
| Preterm CHD N=29 |  |  |  |  |  |  |

Significant p-value bolded
